# An Implementation of Patient-Specific Biventricular Mechanics Simulations With a Deep Learning and Computational Pipeline

**DOI:** 10.3389/fphys.2021.716597

**Published:** 2021-09-16

**Authors:** Renee Miller, Eric Kerfoot, Charlène Mauger, Tevfik F. Ismail, Alistair A. Young, David A. Nordsletten

**Affiliations:** ^1^School of Biomedical Engineering and Imaging Sciences, King's College London, London, United Kingdom; ^2^Auckland MR Research Group, University of Auckland, Auckland, New Zealand; ^3^Department of Biomedical Engineering and Cardiac Surgery, University of Michigan, Ann Arbor, MI, United States

**Keywords:** personalised modelling, biventricular mechanics, parameter identification, automatic segmentation, valve landmark identification

## Abstract

Parameterised patient-specific models of the heart enable quantitative analysis of cardiac function as well as estimation of regional stress and intrinsic tissue stiffness. However, the development of personalised models and subsequent simulations have often required lengthy manual setup, from image labelling through to generating the finite element model and assigning boundary conditions. Recently, rapid patient-specific finite element modelling has been made possible through the use of machine learning techniques. In this paper, utilising multiple neural networks for image labelling and detection of valve landmarks, together with streamlined data integration, a pipeline for generating patient-specific biventricular models is applied to clinically-acquired data from a diverse cohort of individuals, including hypertrophic and dilated cardiomyopathy patients and healthy volunteers. Valve motion from tracked landmarks as well as cavity volumes measured from labelled images are used to drive realistic motion and estimate passive tissue stiffness values. The neural networks are shown to accurately label cardiac regions and features for these diverse morphologies. Furthermore, differences in global intrinsic parameters, such as tissue anisotropy and normalised active tension, between groups illustrate respective underlying changes in tissue composition and/or structure as a result of pathology. This study shows the successful application of a generic pipeline for biventricular modelling, incorporating artificial intelligence solutions, within a diverse cohort.

## 1. Introduction

Cardiovascular disease causes changes in cardiac anatomy, structure, and function—all resulting in changes to the active and passive biomechanics of the myocardium. However, it is difficult to assess intrinsic properties from imaging data alone. Patient-specific computational models can be used to simulate cardiac mechanics and measure quantities such as stress and strain and have the potential to augment current steps in therapy planning, allowing clinicians to test devices, such as left ventricular assist devices (Sack et al., 2018b), and therapies, such as septal myectomy (Huang et al., 2021). Personalised models can also be used to create “virtual cohorts,” running large-scale trials on large numbers of realistic heart models in concert with animal and human studies (Peirlinck et al., 2021).

Personalised models have been used to estimate both passive (e.g., Augenstein et al., 2006) and active (e.g., Marchesseau et al., 2013) parameters. Differences between global stiffness parameters have been identified between healthy and diseased cohorts through the use of patient-specific modelling (Hadjicharalambous et al., 2017; Wang et al., 2018). These passive parameters could be used as an additional diagnostic tool or to track disease progression. Additionally, estimation of heterogeneous stiffness parameters demonstrate the feasibility to identify local differences in tissue properties (Balaban et al., 2018), which can give an indication of regional changes. The optimisation of material parameters has been formulated as a nonlinear optimisation problem which aims to minimise an objective function based on the observation error, typically using displacement (Wang et al., 2018), strain (Augenstein et al., 2005; Wang et al., 2009), or geometric metrics (Nasopoulou et al., 2017). Filtering approaches, such as the use of Kalman filters, have also demonstrated robust and accurate estimation of passive parameters in the presence of noise (Xi et al., 2011). Regional contractility parameters estimated in personalised models have been shown to decrease in infarcted regions (Chabiniok et al., 2012). Although personalised modelling has been demonstrated to offer insights into intrinsic properties of the heart in health and disease, key challenges remain including automation of many cumbersome steps in model development as well as integration of key biomechanical information. For example, most studies have utilised manual segmentation for model development. Additionally, many studies developing personalised models have relied on data which is not typically acquired in a clinical scan (e.g., tagged MRI or intraventricular pressure measurements), thus limiting the size of their cohorts.

The process of generating patient-specific models was once a time-consuming task, requiring manual annotation and segmentation of images to construct an accurate geometric model (Heijman et al., 2008). The advent of and advances in machine learning have enabled automation of many of these tasks, with results ranging in accuracy and reliability (Henglin et al., 2017; Leiner et al., 2019). Deep learning is a subset of the machine learning field of techniques focusing on artificial neural networks which are constructed as deeply interconnected neural structures (Zhang et al., 2018). Neural networks have greater capacity to learn more complex problems than other machine learning methods with a greater ability to generalise to unseen data. These can be applied directly to the labelling of anatomical structures by assigning each pixel/voxel of an image a category probability which associates them with one or more structures. This allows the automation of cardiac segmentation such that an entire short-axis cine dataset can be labelled in seconds without manual initialisation or intervention, rather than hours (e.g., Bai et al., 2018; Chen et al., 2020). Cardiac segmentations can then be used to automatically calculate clinical metrics such as ejection fraction and long-axis strain (Ruijsink et al., 2019), and can be combined with other networks to perform disease classification (Martin-Isla et al., 2020) and feature detection (Bizopoulos and Koutsouris, 2018). In addition to expediting segmentation, trained neural networks can also lead to consistent and standardised results improving reliability and reproducibility. Neural network segmentations can then be used to automate the process of generating patient-specific geometric models.

In order to extend personalised modelling into the clinical domain, there is a need to develop a robust pipeline to not only generate models for diverse cardiac morphologies, but also to run biomechanical simulations using data acquired within a clinical scan. This study presents an AI-driven pipeline for the development of personalised biventricular mechanical models which were used to simulate passive and active mechanics. Novel boundary conditions, driven by neural network derived landmarks, were used to constrain valve motion and cavity volumes. The pipeline was tested in a diverse cohort which included healthy volunteers, patients with DCM and hypertrophic cardiomyopathy (HCM), using only a short-axis cine stack and three long-axis image planes, equivalent to images that would be collected in a standard clinical MR scan.

## 2. Materials and Methods

For all cases in this study, a balanced steady-state free precession sequence was used to collect cine images at short-axis slice locations and three long-axis imaging planes including two (2CH), three (3CH), and four-chamber (4CH) views. Between 20 and 40 images were acquired per cardiac cycle depending on the individual's heart rate. All images were acquired on a Philips Achieva 1.5 T scanner at St. Thomas' Hospital in London. Written informed consent was obtained from all participants prior to scanning. The study protocols for the DCM patients and healthy volunteers (study number 12/LO/1456) and HCM patients (study number 15/NS/0030) were approved by the London Bridge National Research Ethics Service. This initial study includes a cohort of patients with HCM (*n* = 4), DCM (*n* = 4), and healthy volunteers (*n* = 4). Patients with HCM demonstrated heterogeneous patterns of wall thickening, in keeping with the underlying diagnosis. The entire processing pipeline for each case is shown in **Figure 3**. Each block within the figure will be discussed in greater detail in the following sections.

### 2.1. Neural Network Image Labelling

Cine images were passed to two neural networks. The first of which labelled the left ventricular (LV) blood pool, LV myocardium and right ventricular (RV) blood pool in all short and long-axis images, whereas the second returned labels of 10 valve landmarks identifying leaflet insertion points in all long-axis images.

#### 2.1.1. Cine Image Labelling

Full-cycle three-label segmentation was accomplished using a UNet-derived (Ronneberger et al., 2015; Kerfoot et al., 2018, 2019) neural network. Left-ventricular blood pool, left-ventricular myocardium, and right-ventricular blood pool were identified by this network by analysing each two-dimensional slice from a full short-axis stack individually.

**Figure 1:**
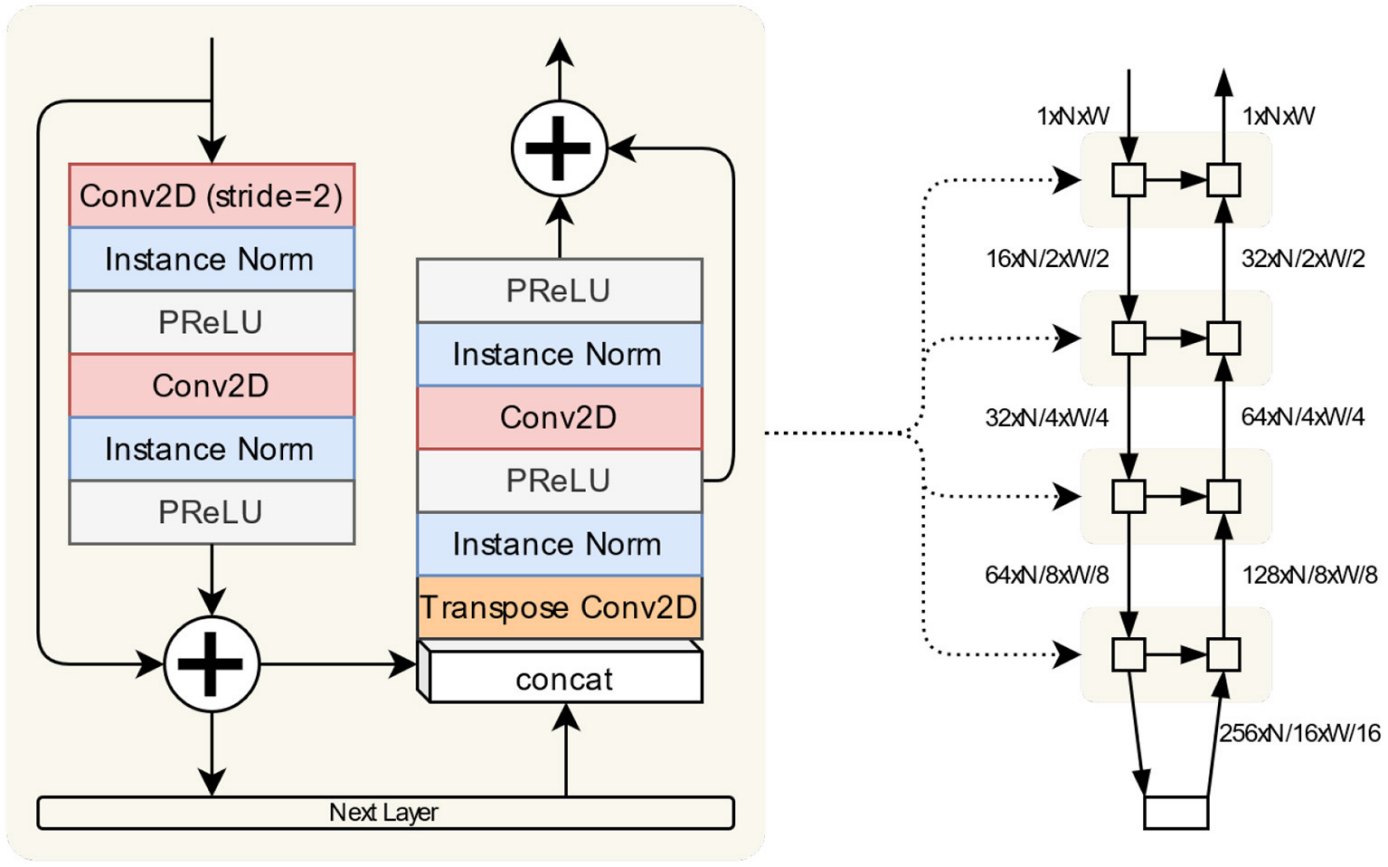
The segmentation network is implemented as a stack of blocks illustrated here. The encode and decode paths along with the skip connection are defined in the same block. The “Next Layer” is either another such block or the bottom encoding block comprised of convolution/normalisation/activation sequences. The overall structure of the network is shown on the right, with the dimensions of tensors passing between layers given relative to an input of shape (1, N, W).

The network architecture is composed of a stack of blocks incorporating the encode and decode paths of the UNet structure (Figure 1). Data flows through the encode side on the left where it passes through a residual unit (He et al., 2016) of convolution/normalisation/activation layers. The output from this unit passes to the next layer in the stack, which is either a further layer of such encode/decode pathways or a final residual unit. The data from the encode path is concatenated with the output from the layer below before being passed through another residual unit in the decode side.

The dataset used for training consisted of 9,095 segmented MR short-axis images (Kerfoot et al., 2019). These were derived from the ACDC challenge dataset (Bernard et al., 2018) of 100 cases and 175 UK Biobank healthy cases. Of the latter, an expert clinician at St. Thomas' Hospital in London segmented 100 healthy cases, 50 cardiomyopathy cases, and 25 randomly selected cases that exhibited sufficient image quality for use as input. Additionally, 215 cases were acquired on a 1.5 T Philips Ingenia scanner at St. Thomas' Hospital in London, and 116 cases from a Siemens Trio 3T scanner (Siemens Healthineers, Erlangen, Germany), and were also segmented by an expert clinician at St. Thomas' Hospital. These cases consisted of healthy volunteers, HCM patients, and patients with cardiac resynchronisation therapy (CRT).

The network was trained for 10,000 iterations. For each iteration, a mini-batch was created by selecting 250 randomly selected images from the dataset. A random selection of flip, transpose, 90° rotation, shift and non-rigid deformation operations were applied to the image and segmentation pairs. The loss function used was a simple Dice loss (Dice, 1945).

#### 2.1.2. Valve Landmark Identification

Landmark coordinates in the three long-axis views were used to identify the locations of the leaflet insertions into the myocardium. Ten landmark locations in total were estimated: six mitral valve locations (two from each view), two aortic locations in the three-chamber view, and two tricuspid locations in the four-chamber view. These landmarks were estimated using a convolutional neural network implemented as a regression from two-dimensional images to a landmark coordinate array (Kerfoot et al., 2021). See **Figure 3**: *Valve Landmark Identification*. Briefly, the network was trained on 8,574 long-axis images collected from HCM (*n* = 3,069) and myocardial infarction (MI, *n* = 5,505) patients. Further details of the dataset used for training can be found in Kerfoot et al. (2021).

**Figure 2:**
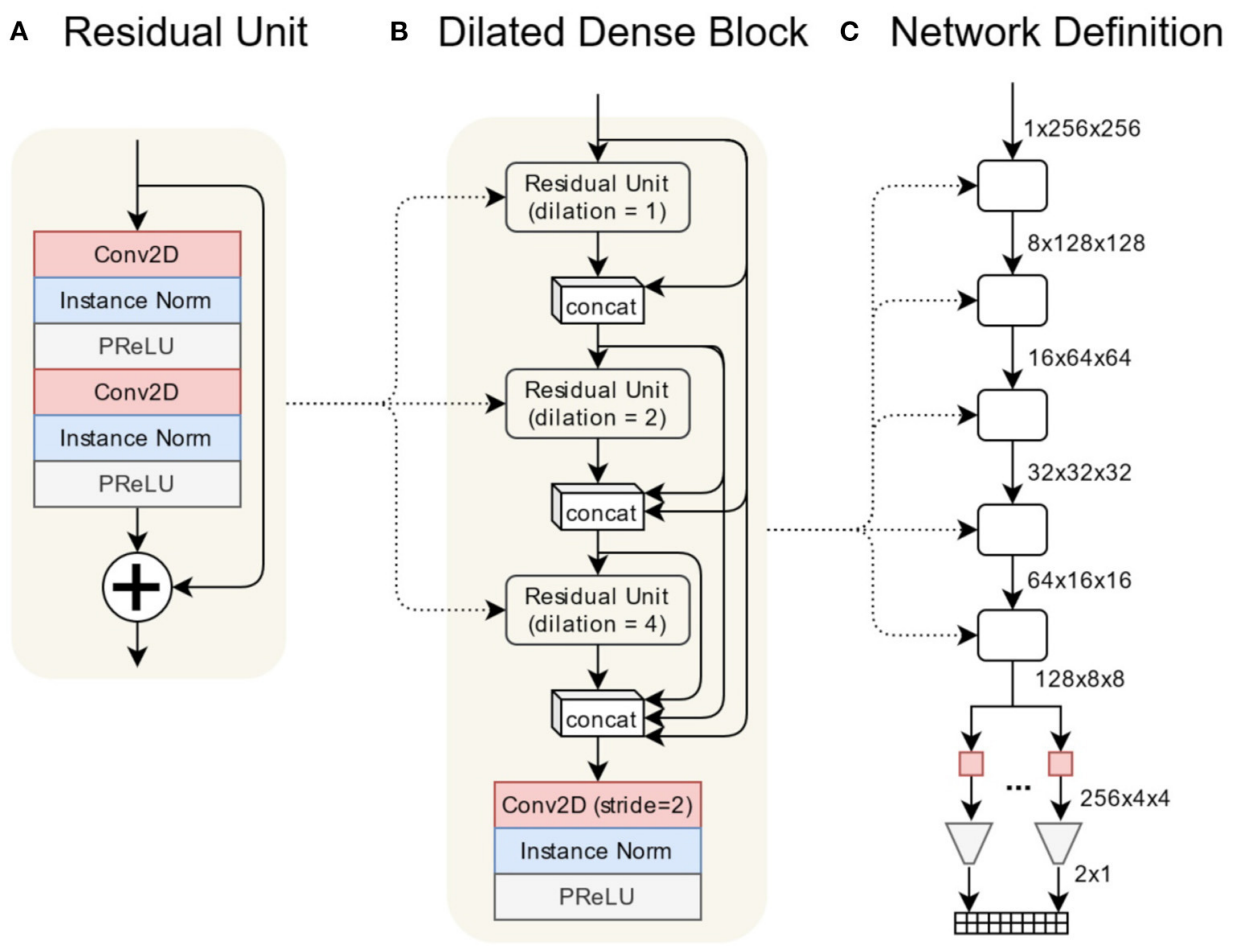
The valve estimation network is composed primarily of a series of densely-connected convolutional layers. Each dense block is composed of three residual units containing 2D convolutions using progressively larger dilation rates. A final convolution reduces the spatial dimension of the volume by 2. The regression network is implemented as a sequence of densely-connected blocks followed by a series of small fully-connected networks relating the final output volume to each landmark coordinate. **(A)** Residual unit, **(B)** dilated dense block, **(C)** network definition.

Figure 2C illustrates the general architecture of the network composed of a sequence of densely-connected blocks of convolutions. The output data from these blocks is then passed to a series of small neural networks trained to recognise the 10 different landmark coordinates. Having condensed the information from the input image to a deep representation, each sub-network learns to recognise which view is represented and determine a location from this representation. From each long-axis image, all ten landmarks are identified. However, the landmarks which do not occur in the input image are inferred to be in the top-left corner at coordinate [0,0].

Figures 2A,B illustrates the architecture of the densely-connected blocks (Huang et al., 2017). Within each block is a residual unit composed of two sets of convolution/normalisation/regularisation layers. The dilation value for the convolutions increments for each succeeding unit, which allows convolutions to recognise features of different scales in the input volume. The output from each unit is concatenated with outputs from previous units. This combined volume is used as the input to the next unit. All such outputs, plus the original input, are concatenated into the final output volume. A final convolution/normalisation/regularisation reduces the output volume in the spatial dimensions by a factor of two.

During training, data augmentation was applied to the images from the manually-annotated dataset. A randomised combination of flip, transpose, zoom, rotate, shift, and non-rigid deformation operations were applied to the image and ground-truth landmark pairs to be fed into the network during training. The images were further augmented with added noise, smooth image intensity variation and k-space dropout to simulate a poor-quality acquisition. The objective of these augmentations was to vary the data the network is trained with to reduce overfitting and improve its generalisation to unseen image types.

#### 2.1.3. Label Quality Control

For the short and long-axis segmentations, labels were cleaned (a) by removing labelled regions with fewer than 50 pixels, disregarding improperly labelled “islands” far from the heart as well as (b) filling holes in the labelled regions. Since valve landmarks were identified for each 2D long-axis image independently (not incorporating temporal continuity), an additional step was implemented to automatically identify landmarks which were incorrectly labelled in order to omit these points. Then, a linear interpolation step was used to interpolate missing points before applying a low-pass filter to temporally smooth landmark displacements. Valve landmarks were used both as input to the model fitting step as well as boundary conditions to constrain valve annuli motion throughout simulations of the cardiac cycle.

### 2.2. Segmentations to Models

Short-axis alignment was performed using the IRTK toolbox (Schnabel et al., 2001) which applies a rigid transformation to individual short-axis planes in order to optimise the overlap between short-axis masks and a model template. Starting with the rigid registration tool's default initialisation, the short-axis images are rigidly moved in the in-plane dimension to account for misalignment during acquisition.

Subsequently, long-axis images were rigidly registered to the short-axis aligned images using the rigid registration algorithm in the IRTK package, also using the tool's default initialisation. Dice scores along the line of intersection between each short and long-axis mask were used to (a) automatically determine which short-axis slices would be used for the model fitting by omitting slices with a dice score <0.5 and (b) to assign weights to each contour point based on their overlap with other data. In this way, long-axis slices which were poorly registered to the short-axis data, even after running the registration step, did not skew or greatly impact the final fitted model.

A biventricular template was then fitted to the segmentations using the two-step iterative method developed in Mauger et al. (2018). In order to do this, contours were automatically generated from the short and long-axis labels (i.e., LV endocardium, RV septum, RV free wall, epicardium, RV insertion points, apex, etc.) in order to fit model surfaces to the contour points. Locations of the mitral, tricuspid, and aortic valve annuli were obtained from the annotated valve landmarks. Due to a lack of segmentation of the RV myocardium, RV epicardial contours were automatically generated by projecting the RV free wall contours in the normal direction at a distance of 3 mm. Briefly, a series of stiff linear least squares fits with a high D-affine regularisation weight was performed to provide an adequate first solution. For each iteration, the Jacobians on 4 × 4 × 4 Gaussian quadrature points were calculated. If all were positive, the model was updated, the regularisation weight was decreased and another iteration was performed. If not, the model was not updated and another optimisation step was performed using diffeomorphic constraints based on the magnitude of the displacement. Models were fit to segmentations at all frames of the cardiac cycle. Surface meshes were used to construct cavity volume curves throughout the cardiac cycle as well as quantify metrics such as wall thickness and ejection fraction.

**Figure 3:**
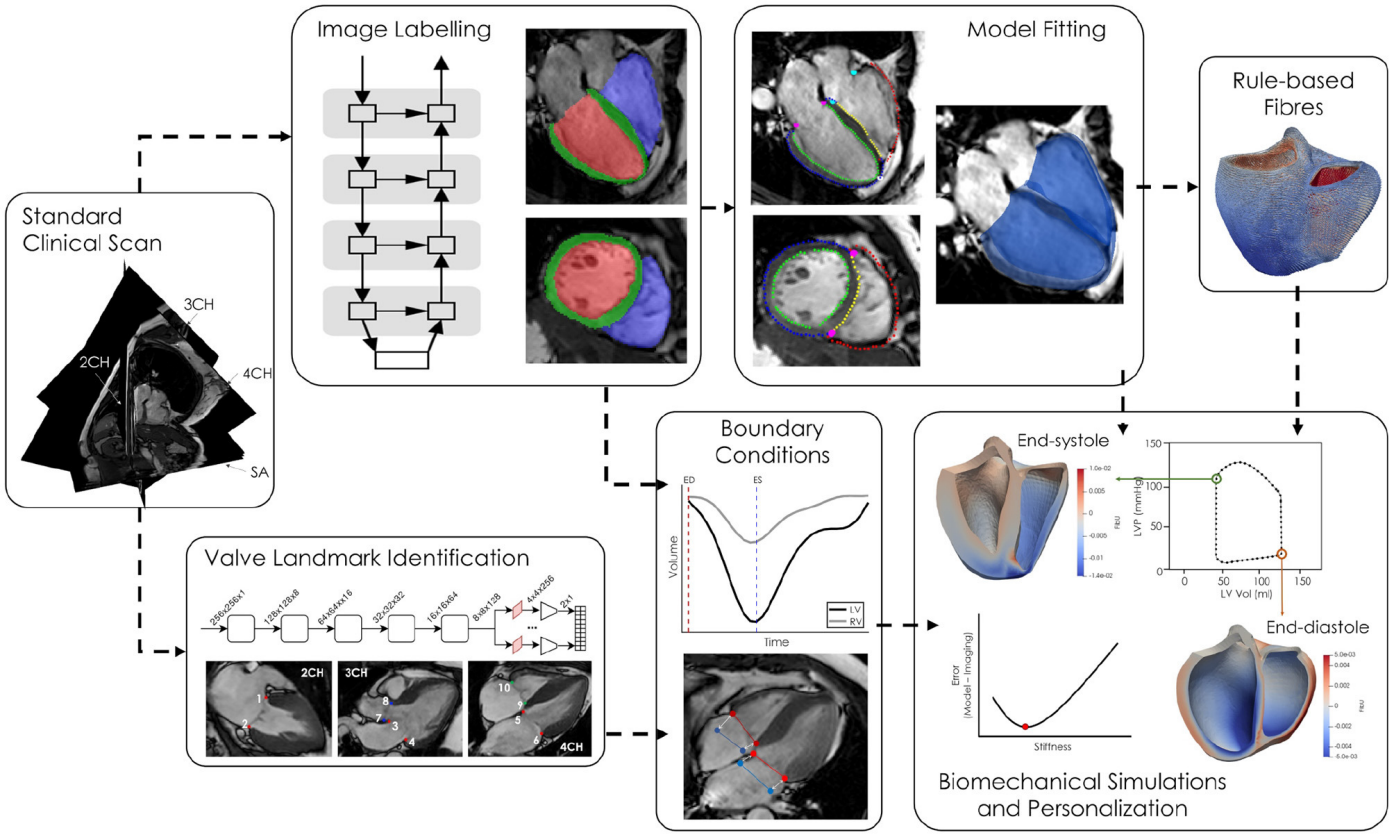
Short and long-axis cine MR images are simultaneously fed into two neural networks, one for labelling the LV blood pool (red), LV myocardium (green), and RV blood pool (blue) and the second labelling ten valve landmarks throughout the cardiac cycle. The segmentations are converted to labelled contours and a biventricular template surface mesh is fitted to the labelled contours. Volumes, derived from the network generated cavity labels, as well as valve annuli motion are used as boundary conditions in the biomechanical simulations. Passive parameters are optimised by minimising the difference between the model and imaged geometries at end-diastole.

From the fitted surface meshes, tetrahedral meshes were generated for the end-systolic time point using SimModeler (Simmetrix^1^). Mesh metrics, including number of nodes and element quality, can be found in Supplementary Table 1. Biventricular fibre fields were created using a rule-based method adapted from Doste et al. (2019) and Bayer et al. (2012). Fibre angles varied from −60 to 60° and −25 to 90° from the epicardium to endocardium in the LV and RV, respectively. Fibre angles at the valve annuli were determined based on high-resolution DTI measurements from *ex-vivo* porcine hearts. Specific angles at each boundary can be found in Supplementary Table 1. An example fibre field can be seen in Figure 3, *Rule-based Fibres*.

### 2.3. Biventricular Modelling

The personalised mechanical models were solved using energy potential minimisation, following (Asner et al., 2017; Hadjicharalambous et al., 2017). In brief, the myocardium is defined by the reference domain $\Omega_{0}\subset {\mathbb{R}}^{3}$ with initial coordinates **X** ∈ Ω_0_. The biventricular domain, Ω_0_, consists of boundaries on the endocardial sides of the LV and RV (denoted $\Gamma_{0}^{lv}$ and $\Gamma_{0}^{rv}$), the wall marking the rings for the mitral ($\Gamma_{0}^{mv}$), aortic ($\Gamma_{0}^{av}$), tricuspid ($\Gamma_{0}^{tv}$), and pulmonary valves ($\Gamma_{0}^{pv}$), as well as the epicardium ($\Gamma_{0}^{epi}$). The orientation of local tissue microstructure across the myocardial wall is given by the fibre, sheet and sheet normal vector fields, (**f**_0_, **s**_0_, **n**_0_). Similarly, at each valve boundary, a circumferential vector field is defined (denoted ${\text{f}}_{0}^{k}$ for $\Gamma_{0}^{k}$, k∈V={mv,av,tv,pv}) which describes the local orientation of connective tissue that comprises each valve orifice. Finally, to enable variations between the LV/septum and the RV, we define a labelling field, ϕ, where ϕ = 1 in the LV/LV septum and ϕ = 0 in the RV/RV septum.

For simulating myocardial function, imaging data is extracted to describe functional changes through time. The change in LV and RV luminal volumes is extracted from images and interpolated to provide ${\left\{V_{k}\left(t\right)\right\}}_{k\in E}$ describing the mean volume trace as computed over a truncated region of each endocardial lumen, E={lv,rv}. The truncation planes are similarly defined by normal vectors ${\left\{{\text{n}}_{k}\left(t\right)\right\}}_{k\in E}$ across both LV and RV lumens. The pressure is given over the cardiac cycle by ${\left\{P_{k}\left(t\right)\right\}}_{k\in E}$ and can be defined either via invasive measures, coupled via a full-circulation model (Arts et al., 2005), or estimated from noninvasive data (Asner et al., 2015). Finally, the motion of each valve plane is encapsulated by the estimated motion of the centre of mass, ${\left\{{\text{u}}_{com}^{k}\left(t\right)\right\}}_{k\in V}$, interpolated over time for each valve, V={mv,av,tv,pv}.

As the biventricular model deforms, the physical domain at time *t*, Ω(*t*), is described using coordinates of its current position **x** = **X** + **u**(*t*), where **u** denotes displacement. Typically the displacement is used to describe the deformation gradient tensor **F** = ∇_0_**u** + **I**, its determinant *J* = det**F** > 0, as well as the material stretch described by the right Cauchy-Green strain tensor **C** = **F**^*T*^**F**. The displacement of the heart is solved by considering either the quasi-static (Asner et al., 2017) or dynamic (Chabiniok et al., 2012; Sermesant et al., 2012) principle of virtual work, with the additional state variables of pressure (*p*), activation state in LV / RV (α_*lv*_, α_*rv*_), and the forces present at each valve plane (${\left\{\lambda^{k}\right\}}_{k\in V}$). In this study, at each time point, *t* ∈ [0, *T*], we seek to find the state variables u(t)∈U, p(t)∈P, α_*lv*_(*t*), α_*rv*_(*t*) ∈ ℝ, and **λ**^*mv*^(*t*), …**λ**^*pv*^(*t*) ∈ ℝ^3^ satisfying the quasi-static virtual work equation,


(1) $$\begin{array}{l} \int_{\Omega_{0}}P_{myo}:\nabla_{0}w+q(K[J-1+k\ln J]-p(t))d\Omega_{0}\\ +\sum_{k\in V}\int_{\Gamma_{0}^{k}}P_{valve}^{k}:\nabla_{0}wd\Gamma_{0}\\ +\sum_{k\in ℰ}\int_{\Gamma_{0}^{k}}P_{k}(t)JF^{-T}N\cdot wd\Gamma_{0}\\ +\sum_{k\in V}q_{k}\left(\int_{\Gamma_{0}^{k}}I_{b}^{k}(t)(u(t)+X)\cdot JF^{-T}Nd\Gamma_{0}-V_{k}(t)\right)\\ +\sum_{k\in V}\int_{\Gamma_{0}^{k}}\lambda^{k}(t)\cdot w-q^{k}\cdot (u_{com}^{k}(t)-u(t))d\Gamma_{0}=0,\\ \forall \;w\in U,q\in ,q_{lv},q_{rv}\in {\mathbb{R}}^{2},\text{ and }q^{mv},\ldots q^{pv}\in {\mathbb{R}}^{3}. \end{array}$$

The virtual work equation describes the internal myocardial stresses and balance of volumetric/pressure change (blue term), the stresses induced by the collagenous valve tissue (red term), the internal pressure exerted by the blood (gold term), the constraint on chamber volumes (purple term), and the added forces required to ensure motion of the valve orifices (green). The specifics of these terms are detailed below.

The internal stresses (blue term) are given by the first Piola-Kirchhoff tensor, **P**_*myo*_, which is described by the hyperelastic-strain energy, Ψ, that can be broken into passive, volumetric, and active strain energy components,


$$\begin{array}{r} {\text{P}}_{myo}\left(\text{C},p,\alpha_{lv},\alpha_{rv}\right)=\frac{\partial \text{Ψ}}{\partial \text{F}}=\frac{\partial {\text{Ψ}}_{p}\left(\text{C}\right)}{\partial \text{F}}+\frac{\partial {\text{Ψ}}_{vol}\left(J,p\right)}{\partial \text{F}} \end{array}$$

(2) $$\begin{array}{r} +\frac{\partial {\text{Ψ}}_{act}\left(\alpha_{lv},\alpha_{rv},I_{f}\right)}{\partial \text{F}}, \end{array}$$

where


(3a) $$\begin{array}{l} \frac{\partial \Psi_{p}}{\partial F}=a_{0}\frac{\exp\{b_{0}({\bar{I}}_{1}-3)\}}{J^{2/3}}\left(F-\frac{I_{1}}{3}F^{-T}\right)\\ +\text{ 2}a_{f}{\left(I_{f}-1\right)}_{+}\exp\{b_{f}{\left(I_{f}-1\right)}_{+}^{2}\}\;f⊗f_{0}, \end{array}$$

(3b) $$\begin{array}{r} \frac{\partial {\text{Ψ}}_{vol}}{\partial \text{F}}=pJ{\text{F}}^{-T}, \end{array}$$

(3c) $$\begin{array}{r} \frac{\partial \Psi_{act}}{\partial F}=(\alpha_{lv}\phi +\alpha_{rv}[1-\phi ])\tanh\;\left\{2{\left(\sqrt{I_{f}}-0.8\right)}_{+}\right\}\left(f⊗f_{0}+\frac{1}{3}F\right), \end{array}$$

and **f** = **Ff**_0_ describes the deformed fibre direction. Here the passive component (Equation 3a) follows the reduced form of the Holzapfel-Ogden model (Holzapfel and Ogden, 2009; Hadjicharalambous et al., 2014a, 2017; Asner et al., 2015) adapted for appropriate use within a nearly-incompressible framework (Nolan et al., 2014) (though numerous alternative models exist, see Chabiniok et al., 2016). The parameters *a*_0_ and *a*_*f*_ linearly scale the stiffness of the ground substrate and fibre direction, respectively, and have units of stress whereas *b*_0_ and *b*_*f*_ scale the exponential behaviour of the isotropic and fibre components and are unitless. ${\bar{I}}_{1}$ is the isochoric form of the first invariant of the right Cauchy-Green strain tensor (*I*_1_ = **C** : **I**) and *I*_*f*_ is the fibre pseudo-invariant *I*_*f*_ = **C**:(**f**_0_ ⊗ **f**_0_). To ensure unique parameters, the number of personalised parameters was reduced to two: *a* and *a*_*f*_. The values of *b*_0_ and *b*_*f*_ were each set to 5.0 to ensure physiological pressure-volume response (Hadjicharalambous et al., 2014a).

The resulting stress from volumetric effects (Equation 3b) results from the nearly-incompressible strain energy,


$$\begin{array}{l} {\text{Ψ}}_{vol}\left(J,p\right)=p\left(J-1\right)-\frac{1}{2K}p^{2}, \end{array}$$

which also governs the relation between volume change and hydrostatic pressure (second part of the blue term). The parameter, *K*, denotes the bulk modulus of the tissue (in this study *K* = 1, 000 kPa).

The active stress, given in Equation (3c), defines the amount of contraction as well as the length dependent mechanisms (Kerckhoffs et al., 2003). Here, stresses were also applied both along fibres as well as across fibres based on known myofibre dispersion (Tangney et al., 2013; Krishnamurthy et al., 2016). Note that the active scalings, α_*lv*_, α_*rv*_, are dotted with the region identifier, ϕ, in order to selectively activate LV and RV chambers. While regional activation can be defined based on eikonal activation times (Tomlinson et al., 2002) or monodomain/bidomain simulations (Potse et al., 2006); here, the contraction of the chambers was approximated by uniform contraction parameters, α_*lv*_ and α_*rv*_.

Additional stresses were added along the surface of each valve orifice (red term), reflecting the fact that each valve annulus is comprised of thin cartilaginous tissue (Hamdan et al., 2012; Gunning and Murphy, 2014). This tissue, comprised of circumferential collagen fibres, is extremely flexible but exhibits strong resistance to annular dilation (e.g., stretch of the collagen fibres). This was incorporated into the model by adding stresses, ${\text{P}}_{valve}^{k}$, applied over each annular plane, $\Gamma_{0}^{k}$, where


(4) $$\begin{array}{r} {\text{P}}_{valve}^{k}\left(\text{C}\right)=\frac{\partial {\text{Ψ}}_{valve}^{k}}{\partial \text{F}}=c_{1}\left(\exp\left\{c_{2}\left(I_{f}^{k}-1\right)\right\}-1\right){\text{f}}^{k}⊗{\text{f}}_{0}^{k}. \end{array}$$

Here, ${\text{f}}^{k}=\text{F}{\text{f}}_{0}^{k}$ describes the deformed circumferential direction of collagen fibres in the *k*^*th*^−annulus and $I_{f}^{k}=\text{C}:\left({\text{f}}_{0}^{k}⊗{\text{f}}_{0}^{k}\right)$ is the pseudo-invariant along collagen fibres. As the stresses induced are exerted along an extremely thin area, and were principally oriented along fibres, the added stresses were incorporated over the annular surfaces. The parameter *c*_1_ accounts for the collagen stiffness scaled by the thickness while the parameter *c*_2_ allows for exponential growth in the fibre stresses. Here, a value of *c*_1_ = 0.1 kPa and *c*_2_ = 0.5 were selected for all valves based on achieving a consistent qualitative annular dilation as typically found *in vivo* and were unchanged across patients (assuming the collagenous structures around the valves was consistent).

Instead of using parameter estimation techniques (Chabiniok et al., 2012; Marchesseau et al., 2013; Asner et al., 2015) to determine the activation of the myocardium, the LV/RV activation was solved for as part of the forward model problem. In this context, the active parameters α_*lv*_, α_*rv*_ act as Lagrange multipliers with the constraint held being that both chambers follow the volume trends observed in the data (purple term). Here, the first term provides the model predicted volumes which must be equal to the volumes prescribed by *V*_*k*_(*t*) (where ${\text{I}}_{b}^{k}\left(t\right)=\left(1/2\right)\beta \left(\text{X}\right)\left(\text{I}-{\text{n}}_{k}⊗{\text{n}}_{k}\right)$ and β(**X**) is a binary variable taking the value of 1 below and 0 above the truncation plane) (Asner et al., 2017). As the pressure at each time point is given (gold term), the active tension scalings are found which enable matching between the model/data. Note, for consistency and stability, the applied pressure *P*_*k*_(*t*) must be greater or equal to the passive pressure at the specified volume *V*_*k*_(*t*).

Valve plane motion was prescribed (green terms) using the valve landmark displacements, ${\text{u}}_{com}^{k}$, extracted from the points predicted by the neural network (discussed in section 2.1). For each valve, the average position over the cardiac cycle was enforced using Lagrange multipliers, **λ**^*k*^. The pulmonary annulus is not visible in any of the long-axis images acquired in this study. It can be viewed in a right ventricular outflow tract (RVOT) view, which is not always acquired in clinical scans. Therefore, in this study, an average displacement, computed from the displacements of the other three valves, was applied to the pulmonary valve. The green terms introduce the multipliers (that can be thought of as reference tractions) which constrain the motion of the centre of mass to move as observed in the data.

Since the unloaded state of myocardium is unknown, the end-systolic geometry was used as the reference geometry. Some studies have used inverse methods to estimate the reference geometry (Krishnamurthy et al., 2013; Wang Y. et al., 2020). These methods are dependent on the choice of material law, constitutive parameters and boundary conditions. An analysis illustrating the impact of these choices and ramifications of boundary conditions is presented in Hadjicharalambous et al. (2021).

Personalised models were solved in a finite element framework with displacements and pressure defined using linear ℙ^1^ elements, see Supplementary Material for further details. Endocardial and valve Lagrange multipliers were scalars. All problems were solved in C**Heart**, a multi-physics finite element solver (Lee et al., 2016).

### 2.4. Diastolic Inflation and Passive Parameter Estimation

Estimation of both *a*_0_ and *a*_*f*_ is not feasible using displacements alone when driving simulations with cavity volumes. However, due to the linear parameter dependence of *a*_0_ and *a*_*f*_ in the reduced Holzapfel-Ogden law, both passive parameters scale linearly with pressure. Therefore, each model was personalised by estimating, γ = *a*_0_/*a*_*f*_, describing the anisotropy of the tissue from displacements and then using an end-diastolic pressure value to obtain *a*_0_ and *a*_*f*_. To do this, simulations of diastolic inflation were first run starting from the end-systolic geometry, prescribing cavity volumes, and valve motion. This was done by solving Equation (1), assuming α_*lv*_, α_*rv*_ were zero, and considering *P*_*lv*_, *P*_*rv*_ as state variables (Asner et al., 2017). In this first phase, the LV/RV volumes were inflated to their end diastolic state, after which the volume was kept constant and values of γ were swept between 1.0 and 0.1. Practically, this was done by setting $a_{0}^{sim}$ and $a_{f}^{sim}$ to 1.0 kPa during inflation. Then, during the sweep, $a_{f}^{sim}$ was kept constant and *a*^*sim*^ was varied. Absolute values of *a*_0_ and *a*_*f*_ were then retrieved by scaling them by the ratio between an end-diastolic pressure value appropriate for each patient group ($EDP_{lv}^{g}$) and the simulated end-diastolic pressure ($p_{lv}^{ED}$). The $EDP_{rv}^{i}$ for each individual case was then found by using the values of *a*_0_ since the stiffness scales with both EDP in the left and right ventricles.


(5) $$\begin{array}{r} a_{0}=a_{0}^{sim}\frac{EDP_{lv}^{g}}{p_{lv}^{ED}}=\gamma a_{f}^{sim}\frac{EDP_{lv}^{g}}{p_{lv}^{ED}},\;a_{f}=a_{f}^{sim}\frac{EDP_{lv}^{g}}{p_{lv}^{ED}},\\ EDP_{rv}^{i}=p_{rv}^{ED}\frac{a_{0}}{a_{0}^{sim}}. \end{array}$$

For each value of γ, the objective function, J was calculated as the root mean square of the distance between contour points obtained from the neural network labels and the deformed model surface, Γ^*lv*^ (see Equation 6). The objective function utilised only contour points from the LV epicardium, LV endocardium and RV septal wall, omitting RV free wall points.


(6) $$\begin{array}{r} J=\sqrt{\frac{1}{N}\overset{N}{\underset{n=1}{\sum }}\underset{\text{x}\in \Gamma^{lv}}{\min}||{\text{x}}_{n}-\text{x}|{|}^{2}} \end{array}$$

Since all data used were acquired from a standard clinical scan, no catheter pressure measurements were acquired. Filling pressures have previously been estimated from the E/A ratio measured from echocardiography (Nagueh et al., 1997) which requires blood flow measurements through the mitral valve. In the absence of echo and 4D flow MRI data, end-diastolic and end-systolic pressure values were found from literature in studies which obtained invasive catheter pressure measurements from within the LV in each patient group (see Table 1). Additionally, normal end-diastolic and end-systolic pressures were taken from Klingensmith et al. (2012). Taking the mean (weighted by sample size) of pressure values from literature, LV end-diastolic pressures ($EDP_{lv}^{g}$) were assigned to be 8 mmHg (1.1 kPa), 20.2 mmHg (2.7 kPa), and 24.2 mmHg (3.2 kPa) for the healthy volunteers, DCM patients and HCM patients, respectively. Similarly, $ESP_{lv}^{g}$ values were set to 120.0 mmHg (16.0 kPa), 120.0 mmHg (16.0 kPa), and 183.1 mmHg (24.4 kPa) for each group, respectively. A representative pressure trace (Russell et al., 2012) was scaled to group pressure values at end-diastole and end-systole for both the LV and RV. Then, each segment of the pressure trace (i.e., ED to eIVC, eIVC to ES, ES to eIVR, eIVR to diastasis, and diastasis to ED) was temporally scaled for each individual based on valve opening and closing times in the cine images.

**Table 1:** Left ventricular pressure measurements from literature denoting mean pressure ± one standard deviation as well as sample sizes in each study.

**References**	**Group**	**Mean ± Std (mmHg)**	**Sample size (*n*)**
**End-diastolic pressures**			
Opherk et al. (1983)	Idiopathic DCM	18.6 ± 11.4	12
Kass et al. (1999)	DCM	24.8 ± 7.8	18
Hayashida et al. (1990)	DCM	14.0 ± 10.0	17
Nagueh et al. (2005)	HCM	23.0 ± 6.0	35
Nishimura et al. (1996a)	HCM	25.0 ± 9.0	54
**End-systolic pressures**			
Romeo et al. (1989)	Idiopathic DCM	120.0 ± 20.0	69
Nishimura et al. (1996a)	HCM	183.0 ± 42.0	54
Nishimura et al. (1996b)	Obstructive HCM	196.0 ± 43.0	21
Nishimura et al. (1996b)	Non-obstructive HCM	150.0 ± 29.0	8

## 3. Results

### 3.1. Neural Network Segmentation and Landmark Labelling

**Figure 4:**
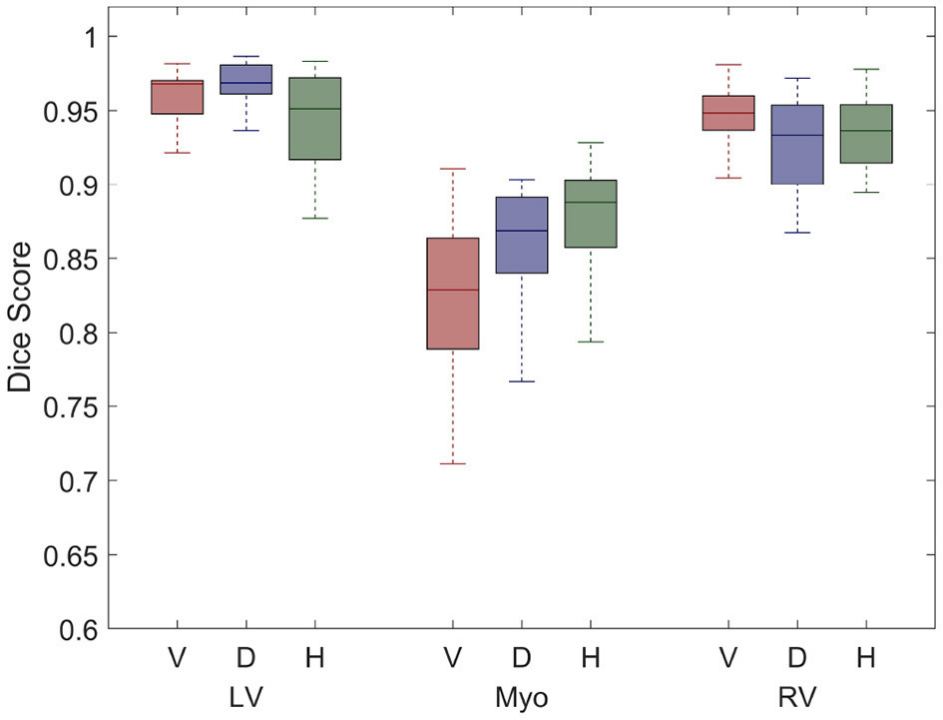
Boxplots illustrate dice scores for each label: LV blood pool (LV), LV myocardium (Myo), and RV blood pool are shown for the healthy volunteers (V), DCM patients (D), and HCM patients (H). The centre line of each boxplot represents the median and the whiskers denote the 25th and 75th percentiles.

All short and long-axis images were manually segmented at the end-diastolic state in order to measure accuracy of the network. Boxplots in Figure 4 plot dice scores measuring similarity between manual and neural network segmentations for each label and group. Results show that the largest errors occur in the segmentation of the myocardium, with HCM cases having the lowest dice scores for this label.

**Figure 5:**
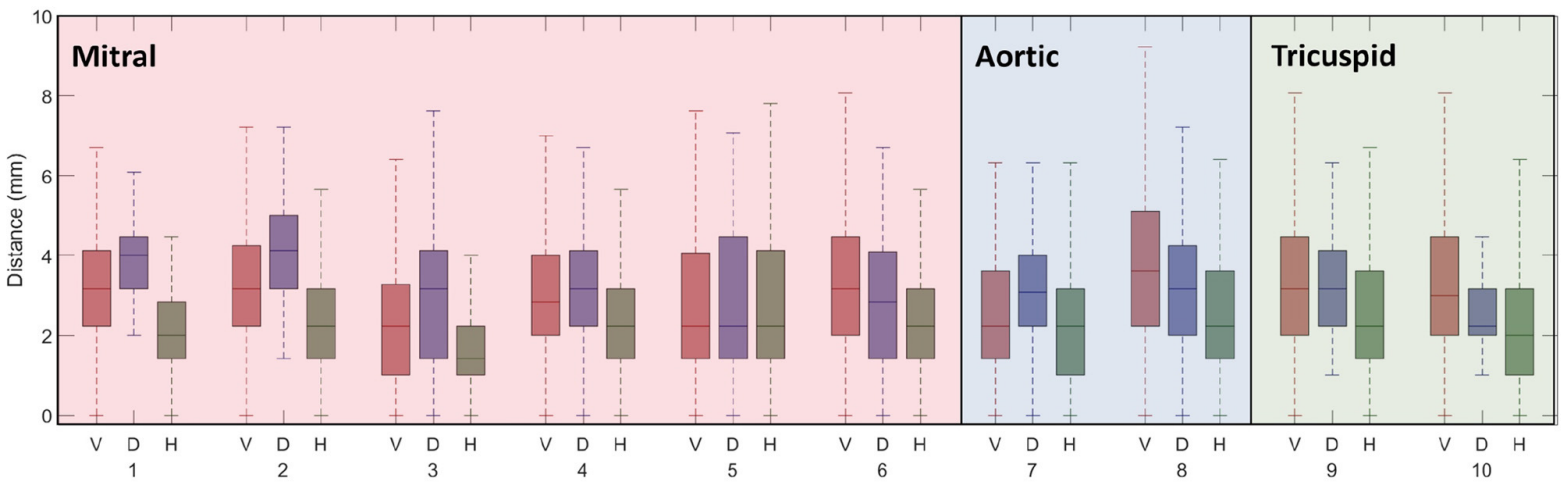
Boxplots of valve annotation errors for all 10 valve landmarks are shown for selected healthy volunteers (V), DCM patients (D), and HCM patients (H) in which valve landmarks were manually identified over the entire cardiac cycle. Each boxplot represents errors throughout the cardiac cycle. The centre line of each boxplot represents the median and the whiskers denote the 25th and 75th percentiles. The 10 valve landmarks correspond to those shown in Figure 3, *Valve Landmark Identification*.

Errors (in mm) between predicted and manually annotated valve landmarks are shown in Figure 5 and were generally within 3 mm of the manually annotated position (~2 pixels). Six landmarks are labelled for the mitral valve (in the 2CH, 3CH, and 4CH images) whereas only two landmarks are labelled for the aortic and tricuspid valves each. Generally, landmarks were more accurately identified in the HCM group.

### 3.2. Model Fitting and Geometric Measurements

**Figure 6:**
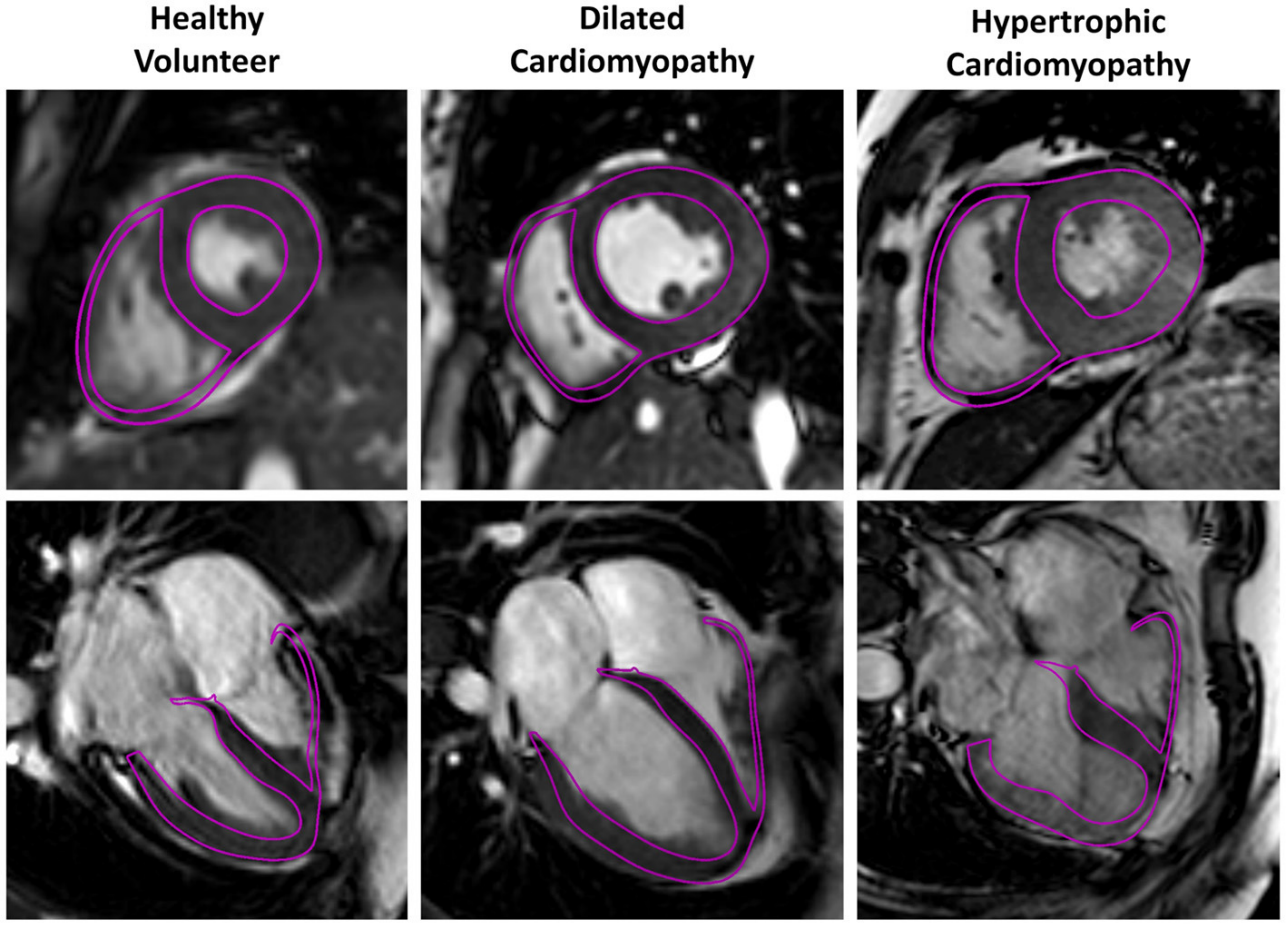
Three representative cases for the healthy volunteer, DCM and HCM groups are shown below with models fit to the neural network segmentations. Model surfaces at end-systole (purple) are overlayed on a single long-axis and short-axis image.

The model fitting algorithm was able to accurately represent the various morphologies within the diverse cohort. Figure 6 illustrates models fit to a healthy volunteer, a DCM patient and an HCM patient with septal hypertrophy at the end-systolic time point. Clinical metrics, such as end-diastolic volumes, end-systolic volumes and wall thickness are reported for each case in Table 2. Compared to the healthy volunteers, the DCM cases have a higher mean LV end-diastolic (EDV) and end-systolic volume (ESV). Conversely, HCM patients have both reduced EDV and ESV in the left ventricle. Healthy volunteers and HCM patients demonstrate LV ejection fractions (EF) in a normal range (50% < EF < 70%) whereas DCM patients exhibit a depressed EF by definition. In all groups, RVEF values fell between (37.9% < EF < 55.9%) without discernible differences between group means. Wall thickness was greatest in the HCM cohort and showed minimal changes in the DCM group between ED and ES.

**Table 2:** Functional and geometric indices: end-diastolic volume (EDV), end-systolic volume (ESV), ejection fraction (EF), wall thickness (WT).

	**Left ventricle**					**Right ventricle**		
**Case**	**EDV (mL)**	**ESV (mL)**	**EF (%)**	**WT_***ED***_ (mm)**	**WT_***ES***_ (mm)**	**EDV (mL)**	**ESV (mL)**	**EF (%)**
**Healthy volunteers**								
v1	172.7	80.8	53.2	7.2	8.7	104.2	64.2	38.4
v2	169.9	79.4	53.3	6.3	8.3	114.4	48.9	57.3
v3	170.1	76.7	54.9	7.2	8.8	104.9	47.6	54.6
v4	123.7	48.6	60.7	6.4	7.9	81.1	42.0	48.2
Mean	159.1	71.4	55.5	6.8	8.4	101.1	50.7	49.6
Std	23.7	15.3	3.5	0.5	0.4	14.2	9.5	8.4
**DCM patients**								
d1	124.4	73.7	40.8	7.4	8.9	57.9	36.4	37.1
d2	231.8	130.0	44.0	8.8	9.0	106.8	57.8	45.9
d3	172.7	90.1	47.8	7.7	9.1	126.5	58.4	53.8
d4	171.0	99.9	41.5	10.0	9.6	87.8	46.4	47.2
Mean	175.0	98.4	43.5	8.4	9.1	94.7	49.8	46.0
Std	44.0	23.6	3.2	1.2	0.3	29.2	10.5	6.9
**HCM patients**								
h1	125.8	59.2	52.9	11.7	13.4	69.9	34.4	50.8
h2	105.8	35.1	66.8	9.1	9.8	65.2	31.7	51.4
h3	134.9	55.3	59.1	9.2	11.4	87.0	44.1	49.3
h4	113.7	41.0	63.9	9.5	11.6	98.4	44.8	54.5
Mean	120.0	47.6	60.7	9.8	11.5	80.1	38.7	51.5
Std	12.9	11.5	6.1	1.2	1.5	15.4	6.7	2.2

### 3.3. Passive and Active Parameterisation

The fibre stiffness ratio, γ, was estimated for all cases using parameter sweeps and the optimal values are listed in Table 3, along with the end-diastolic pressure values used to scale *a*_0_ and *a*_*f*_ to meaningful stiffness estimates. Values of γ close to 1 indicate that the material is more isotropic whereas a value of 0.1 would indicate a highly anisotropic material. The value of γ also influences the final inflated geometry where larger values result in a more spherical shape. The mean value of γ for the volunteers is less than that for the DCM (*p* = 0.2) and HCM (*p* = 0.05) patient groups, indicating that healthy myocardium may be slightly more anisotropic in this small cohort. Additionally, larger γ values are in line with more spherical shapes observed in DCM hearts.

**Table 3:** Personalised passive and active parameters.

**Case**	**γ**	**LVEDP (kPa)**	***a*_0_ (kPa)**	***a*_*f*_ (kPa)**	**LVESP (kPa)**	** $\alpha_{lv}^{max}$ **	** ${\hat{t}}_{lv}^{max}$ **	**RVESP (kPa)**	** $\alpha_{rv}^{max}$ **	** ${\hat{t}}_{rv}^{max}$ **
**Healthy volunteers**										
v1	0.31	1.07	0.27	0.86	16.0	248.1	0.75	4.0	129.9	0.69
v2	0.49		0.20	0.41		236.8	0.75		124.4	0.83
v3	0.34		0.32	0.94		271.9	0.85		204.8	0.77
v4	0.43		0.16	0.38		164.5	0.83		162.5	0.67
Mean	0.39		0.24	0.65		230.3	0.80		155.4	0.74
Std	0.08		0.07	0.30		46.3	0.05		37.0	0.08
**DCM patients**										
d1	0.64	2.69	2.68	4.19	16.0	242.0	0.82	4.0	70.7	0.99
d2	0.44		2.21	5.03		221.8	0.81		303.5	0.63
d3	0.34		1.39	4.071		163.7	0.88		244.4	0.82
d4	0.93		2.17	2.33		295.0	0.83		50.0	0.84
Mean	0.59		2.11	3.91		230.6	0.84		167.1	0.82
Std	0.26		0.54	1.14		54.3	0.03		125.9	0.15
**HCM patients**										
h1	0.64	3.23	1.76	2.74	24.4	303.0	0.70	6.1	173.7	0.83
h2	0.61		0.31	0.50		365.5	0.84		217.7	0.73
h3	0.40		0.61	1.52		278.5	0.83		231.7	0.75
h4	0.58		0.42	0.72		337.1	0.87		200.3	0.75
Mean	0.56		0.77	1.37		321.0	0.81		205.8	0.76
Std	0.11		0.67	1.02		38.2	0.08		25.0	0.05

*γ represents the anisotropic stiffness ratio (a_0_/a_f_). Times to peak activation (${\hat{t}}^{max}$) in the LV and RV are represented as a percentage of the systolic phase*.

**Figure 7:**
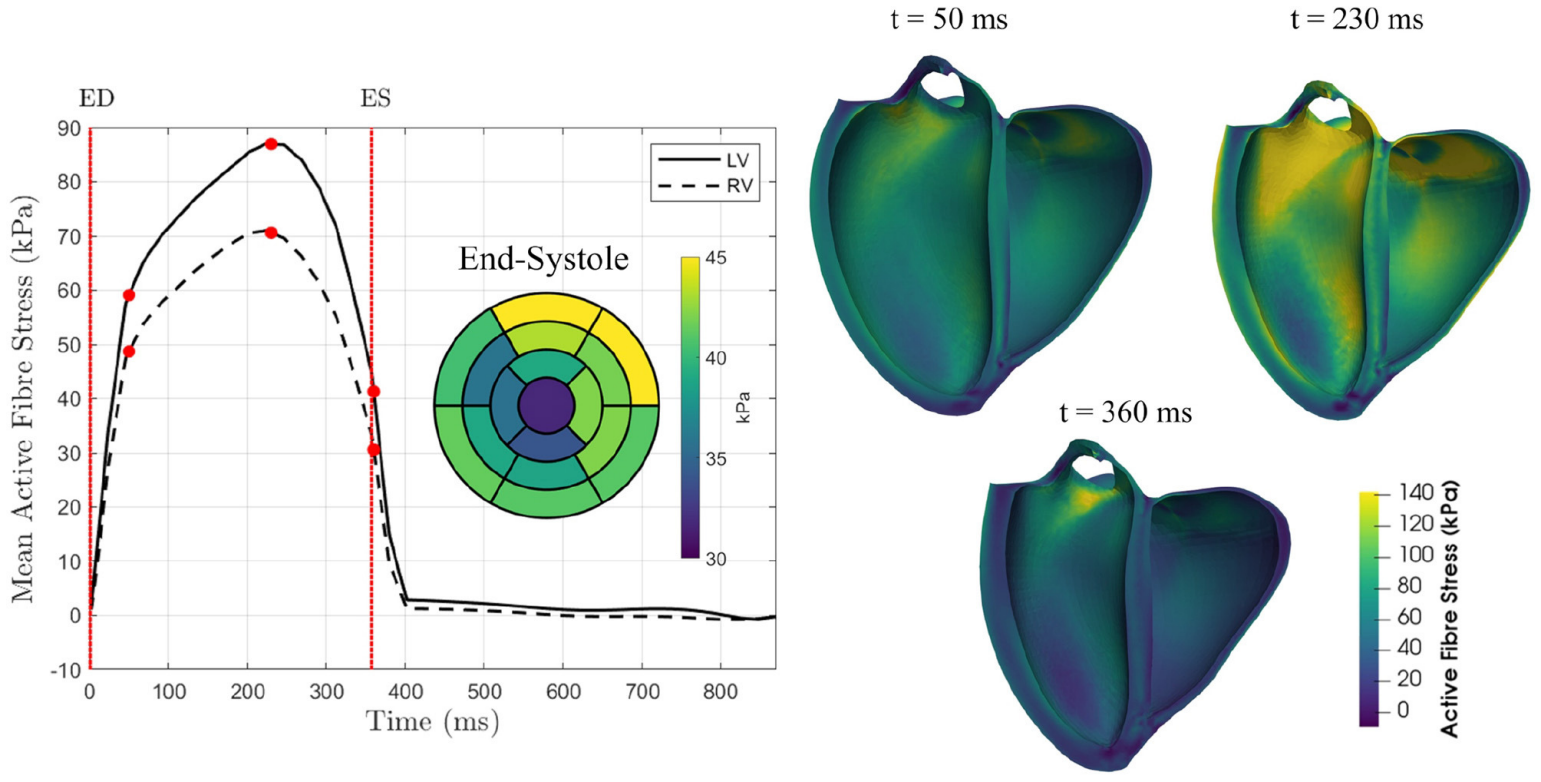
Mean active fibre stress over the cardiac cycle in both the LV and RV for a single case (v1) illustrating the active stress distribution over the entire heart at three points during active contraction: early systole (t = 50 ms), peak active contraction (t = 230 ms), and end-systole (t = 360 ms). The bullseye plot shows the regional distribution of mean active fibre stress over the 17 AHA regions at end-systole.

**Figure 8:**
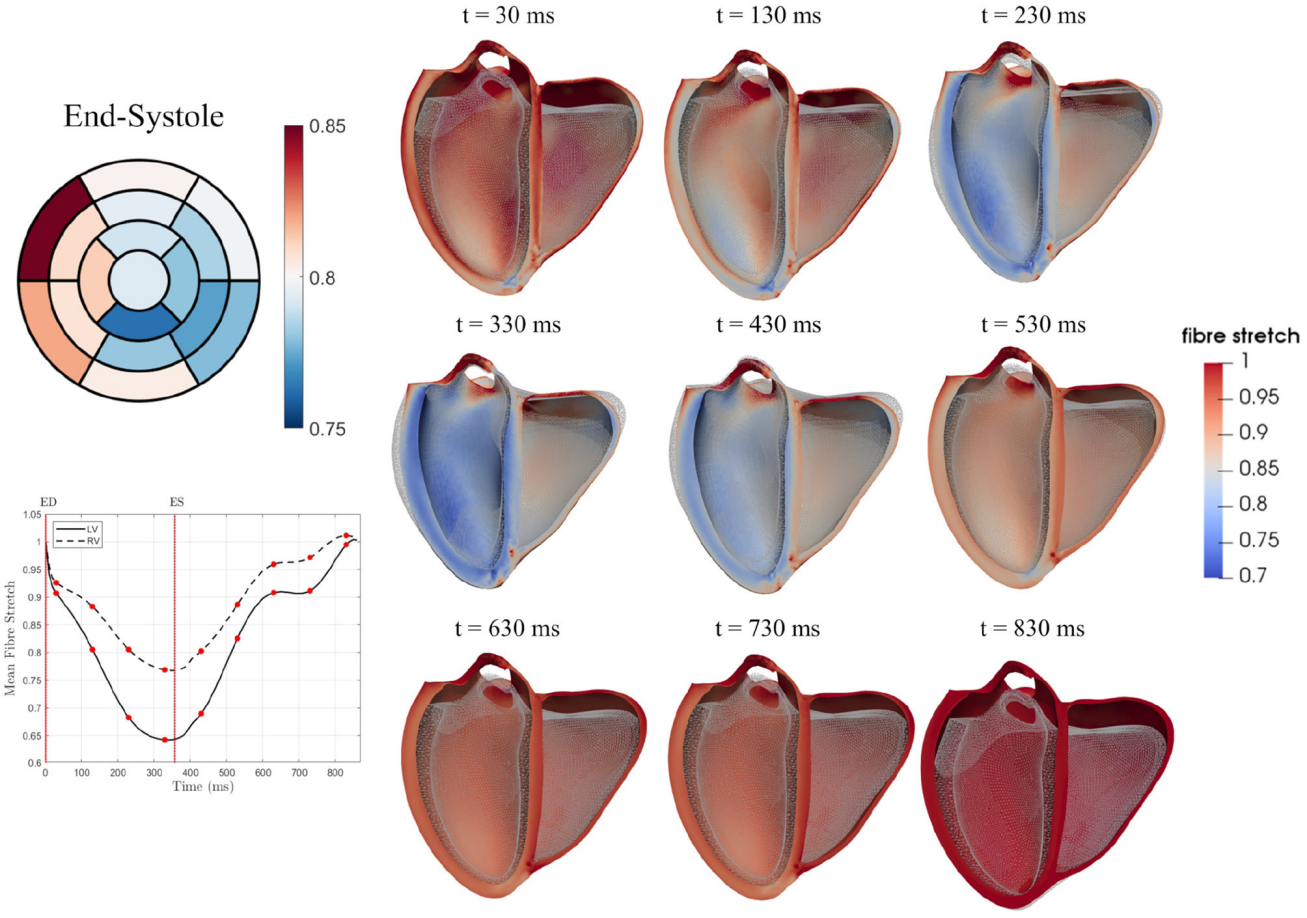
Mean fibre stretch over the cardiac cycle is shown for both the LV and RV for a single case (v1). Fibre stretch over the cardiac cycle is also plotted for nine time points with the reference state model (ES) shown as a wireframe mesh. The bullseye plot illustrates differences in regional stretch at the end-systolic state in the 17 AHA regions.

Two active tension scaling parameters, for the LV and RV, were estimated throughout the cardiac cycle for each case. Normalised time-to-peak (${\hat{t}}_{lv}^{max}$ and ${\hat{t}}_{rv}^{max}$) as well as peak scaling parameters ($\alpha_{lv}^{max}$ and $\alpha_{rv}^{max}$) are listed in Table 3. It should be noted that the traces of α_*lv*_ and α_*rv*_ are dependent on volume changes as well as pressures. Since higher ESP values were assigned in the HCM cases, it can be seen that the peak values of $\alpha_{lv}^{max}$ and $\alpha_{rv}^{max}$ are greater in this group. There were no significant differences between time to peak activation. Figure 7 shows mean active fibre stress over the cardiac cycle in the LV and RV as well as panels showing fibre stress patterns throughout the model for a single case (v1) at three time points during systole. The highest stresses are seen near the base of the left and right ventricles. Fibre stretch with respect to the end-diastolic state is plotted for a representative case illustrating model deformation and regional stretch patterns over the cardiac cycle (Figure 8). A bullseye plot of fibre stretch at end-systole illustrates that the largest values (<0.55) are seen in the LV free wall whereas fibre stretch is restricted in the basal septal region near the valves.

**Figure 9:**
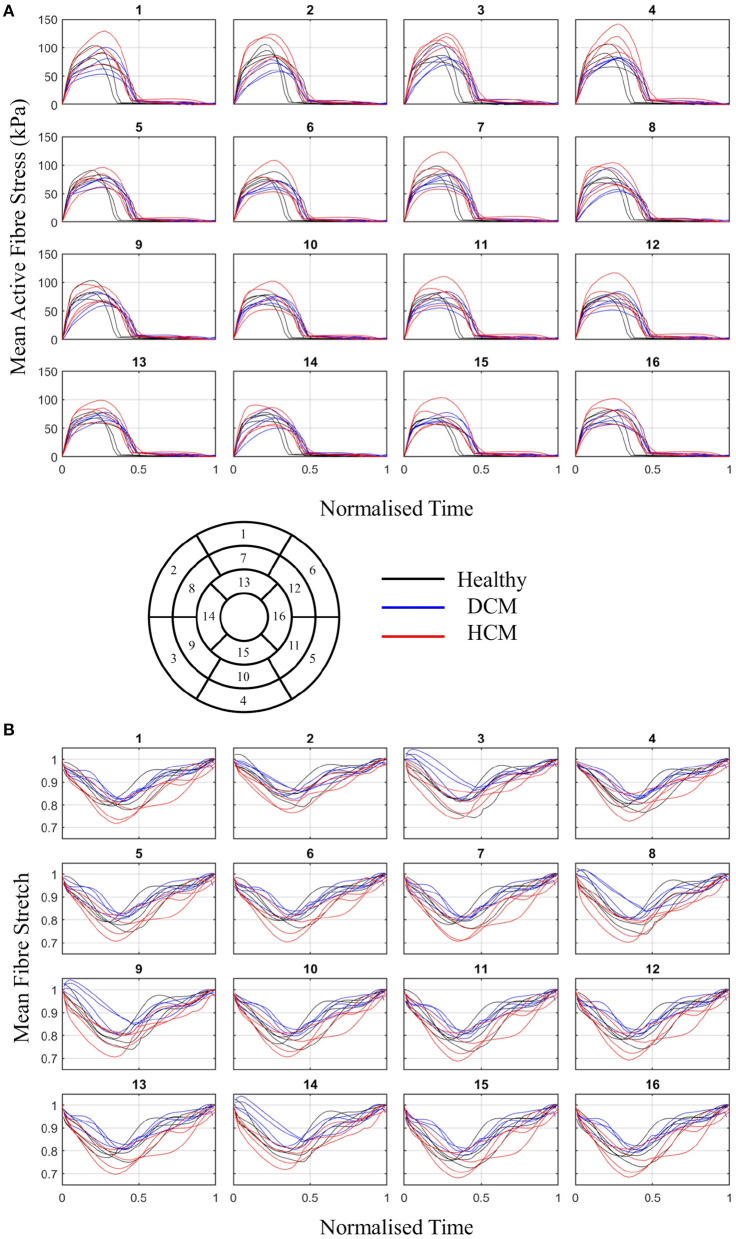
**(A)** Mean active fibre stress and **(B)** fibre stretch over the cardiac cycle in 16 AHA regions of the LV for healthy (black), DCM (blue), and HCM (red) groups.

Both mean active fibre stress and mean fibre stretch in the LV are plotted for 16 AHA segments for all cases in Figure 9, illustrating group differences. Peak fibre stretch is smaller in DCM cases when compared to healthy volunteers in 12 out of 16 AHA regions (*p* < 0.05). Regional fibre stretch demonstrates that, in some DCM cases, some segments exhibit further stretching of fibres (values >1.0) in early phases of systolic contraction. Circumferential and longitudinal stretch, common clinical metrics, are also plotted for 16 AHA segments for all cases in Supplementary Figure 8. The mean for each segment and group are listed in Supplementary Table 4.

## 4. Discussion

The primary goal of this study was to implement a pipeline for running full-cycle simulations using personalised biventricular models generated entirely from neural-network labels. In this process, no manual segmentation was done for the cases presented, other than for analysis of the accuracy of each network. The time and computational resources used for the pipeline are given in Supplementary Table 5 and demonstrate a clear advantage over manual methods. Additionally, all data used in this study was obtained using sequences common to any standard clinical MR scan. Due to its ability to be applied to diverse datasets, this pipeline could be used to develop an *in silico* cohort based on true patient data. This virtual cohort would be invaluable for testing novel therapies and devices alongside human and animal studies. Additionally, personalised metrics obtained from the models (e.g., anisotropy, material stiffness) could be further used to either classify patients or mark disease progression. However, larger sample sizes are needed in order to better understand differences between patient classes. Additional data, where available, could be used to augment the robustness of the personalised models, such as the use of tagged MR data for passive parameterisation (Asner et al., 2015). The use of a biventricular template along with fitting weights assigned to contours based on data fidelity enabled the generation of high-quality meshes suitable for biomechanical simulations with minimal user intervention. Neural-network identified leaflet landmarks were used to prescribe average valve motion on each valve in the model, allowing for physiological basal motion of both ventricles.

### 4.1. Neural Networks

The neural network was able to accurately label the left and right ventricles from standard clinical images in a diverse cohort. The network captured the varied morphology of heart shapes in both DCM and HCM patients. Dice scores for labelling the LV blood pool were comparable to those from other segmentation networks (Wang et al., 2021) and the RV dice scores demonstrated greater accuracy than previous studies (Luo et al., 2016; Tran, 2016). However, the largest errors arose in labels of the myocardium. Although a comparison to inter-observer error was not done as part of this study, previous groups have compared annotations from multiple observers using the UK Biobank (Attar et al., 2019) as well as ACDC (Bernard et al., 2018) data sets. Similar to results shown in Figure 4, inter-observer errors for myocardium are greater than those for both the LV and RV blood pools in both data sets. Dice scores observed in this study are higher than the inter-observer dice scores reported for the LV and RV blood pools in Attar et al. (2019). One possible reason for the larger errors in the segmentation of the myocardium could be due to its annular shape which has a larger perimeter. Any equal overlap shifts would produce a greater error when compared to any shift in segmentation of the blood pools.

The second neural network labelled 10 different valve landmarks in each long-axis image to within 2–3 pixels of accuracy. These errors are similar to those encountered using common tracking algorithms and the method does not require manual initialisation (Kerfoot et al., 2021). Of the 10 valve landmarks, four demonstrated lower neural network predicted errors than interobserver errors (Kerfoot et al., 2021). The error can vary considerably throughout the cycle and between patients as each image is treated individually—i.e., no temporal consistency is taken into account in the neural network. There was no single patient that performed worse than others. Higher errors seen in the identification of landmark 8, on the septal side of the aortic valve can be attributed to image artefacts during systolic blood flow through the aortic outflow tract. Although improvements can be made in future work to increase the accuracy of both neural networks, the study focused on demonstrating their utility in driving model generation and biomechanical personalisation for a diverse set of patients.

**Figure 10:**
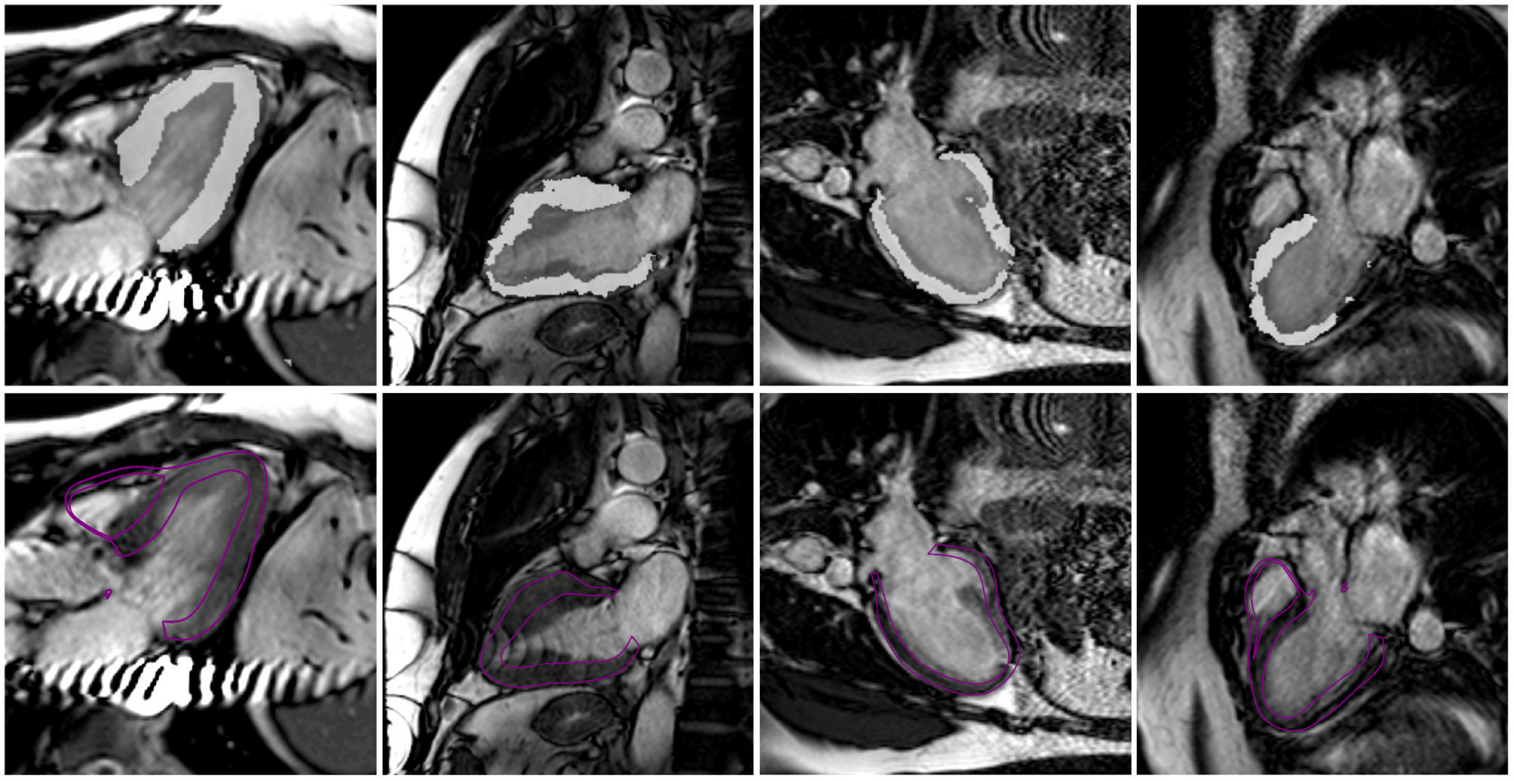
Selected long-axis images which were part of the 12 cases (h1, h4, d2, d4) used in this study which demonstrate poor image quality due to imaging artefacts. In the first row, the LV blood pool and myocardial segmentations obtained using the neural network are overlain on top of each image to qualitatively show the impact of the image quality on the network segmentation. In the second row, the final fitted model surface is shown on top of the image.

In order to have a pipeline that is robust to the presence of noise and artefacts in the imaging data, the neural network training process introduces noise to the images in various ways (e.g., dropout in k-space) so that it learns to account for the noise it may encounter in the imaging data. Additionally, due to the use of a model template fit to all short- and long-axis contours simultaneously, the pipeline is robust to the presence of a single or even multiple poor-quality images within a dataset. This, however, can result in a smooth surface that does not conform to small features. To demonstrate the robustness of the neural network segmentation and resulting pipeline, selected poor-quality images are shown in Figure 10 which were part of the 12 datasets used in this study. The poor image quality resulted in deteriorated segmentations. However, the final fitted model, which also takes into account all short-axis information, produced an adequate estimate of the long-axis shape. The pipeline could be improved by further augmenting the neural network with images that mimic typical artefacts found in MR images.

### 4.2. Clinical Metrics

Beyond improving the generation of computational models, trained neural networks provide a mechanism for automatically characterising common clinical metrics. In the DCM group, the mean EDV and ESV were greater than those measured in the healthy volunteers. Similarly, the mean LVEF was less than that in both the healthy group, marking the deteriorated contractile and diastolic filling function typically clinically associated with DCM (Rihal et al., 1991). Conversely, the mean EDV and ESV values were slightly smaller in the HCM group when compared to the healthy volunteers. As commonly reported in HCM patients, the LVEF in this group was slightly elevated when compared to the healthy group (Haland et al., 2017). HCM patients exhibited greater wall thickness at both end-diastole and end-systole when compared to both the DCM patients and healthy volunteers. Typically, HCM is characterised by a wall thickness >12 mm during diastole. Although the wall thickness values shown in Table 2 report mean values <12 mm, isolated hypertrophic regions in each patient demonstrate areas of hypertrophy >12 mm. Regional plots of wall thickness averaged over each cohort are shown in Supplementary Figure 4 using the 17-segment AHA model. In each of these three groups, no significant differences were observed in the mean RVEF. However, the HCM patients demonstrated lower values of EDV and ESV in the RV than the other two groups. This pipeline has demonstrated the ability to rapidly generate common clinical metrics such as EF and wall thickness as well as cavity volumes over the entire cardiac cycle without the need for manual processing. Aside from using these values in clinical decision making, they can also be used as input into personalised biomechanical models.

### 4.3. Valve Motion

This study presents a novel means of constraining valve motion. Displacement was prescribed to valve centroids based on the motion of the identified landmarks from the neural network. In other cardiac modelling studies, basal motion is often constrained by restricting longitudinal motion (e.g., Sack et al., 2018b; Finsberg et al., 2019; Wang Z. J. et al., 2020) or applying an average motion measured from imaging data (e.g., Hadjicharalambous et al., 2017). In another study, basal motion was constrained by tethering the pulmonary outflow tract to a fixed point (Sack et al., 2018a). In truncated models, without the inclusion of anatomical landmarks, tagged magnetic resonance imaging (MRI) data is necessary to measure longitudinal motion, which may not be available in all clinical scans. The use of a biventricular model with all four valve annuli along with the neural network-defined leaflet insertion points allowed for the integration of longitudinal motion measured from imaging data into the computational model. Further, to impose a constraint similar to the stiff valve annulus, an additional stiffness term was used to restrict annular dilation.

### 4.4. Model Personalisation

Integration of imaging data with personalised biomechanical models enables estimation of intrinsic material stiffness parameters, providing important information about the mechanical state of the myocardium. In this study, we focused on determination of bulk and fibre material parameters, fit by adjusting their ratio, γ. The mean value of γ, which is independent of pressure, was smaller for the healthy volunteers than those estimated for the DCM and HCM patient groups. These weak differences may indicate that the myocardium in healthy individuals is more anisotropic than in pathological hearts. However, these differences were not statistically significant. A power analysis suggests that using eight samples would enable these differences to reach significance. In order to demonstrate statistically significant differences between α^*max*^ values in the LV for DCM and HCM groups, 11 samples would be needed. To distinguish differences between the time to peak contraction in diseased patients and healthy volunteers, 46 samples are needed. Therefore, future studies will aim to expand the sample size to demonstrate the pipeline's utility in providing metrics which distinguish between patient groups.

Simulation outcomes such as strain are relatively independent of the estimated value of γ since the simulations are driven by cavity volumes. However, stress would be more affected by a change in passive material properties. Fibrosis, common in both DCM and HCM patients (Aurigemma et al., 2006; Marian and Braunwald, 2017), results from the growth of collagen within cardiac tissue and may impact tissue anisotropy. The estimated value of γ is also strongly influenced by the angles defined in the rule-based fibre field (Asner et al., 2015; Hadjicharalambous et al., 2017; Campos et al., 2020). As rapid *in vivo* diffusion tensor MRI sequences improve (Stoeck et al., 2018), personalised fibre fields will augment the robustness of the presented pipeline. Methods of using low-resolution *in vivo* data along with statistical models of population fibre fields may provide a new means of personalisation without significantly adding to the clinical scan time (Stimm et al., 2021).

The objective function used for determining the optimal value of γ utilised LV contour points only. The RV deformation is impacted significantly by epicardial boundary conditions due to its thin wall. Various approaches have been used in previous studies to constrain epicardial dilation, such as a spring force acting in the normal direction (Levrero-Florencio et al., 2020; Strocchi et al., 2020) or parallel spring and dashpot forces (Pfaller et al., 2019). However, there remains a lack of clear consensus on the role of the pericardium in restricting myocardial motion and whether or not the inclusion of epicardial constraints improves model personalisation. Therefore, simulations in this study were run without the addition of boundary conditions on the epicardium. However, without these constraints, the right ventricular deformation did not sufficiently match the imaging data (Supplementary Figure 6). Objective function curves with and without the inclusion of RV free wall points are shown in Supplementary Figure 7. Including the RV in the objective function resulted in larger errors and, in some cases, resulted in curves with no unique minimum. The inclusion of the RV in the mechanics problem, however, plays a vital role in restricting motion of the septum (Hadjicharalambous et al., 2017). In future studies, RV epicardial boundary conditions should be tested which result in accurate RV deformation.

This method also presents an elegant solution for estimating dynamically varying active scaling parameters in both the LV and RV in the forward model problem. It avoids data assimilation methods which often require repeated simulations and thus, the presented method reduces computation time. In some diseased states, contractility can vary regionally over the whole heart, such as the case in patients with a myocardial infarction (Chabiniok et al., 2012). In this case, utilising additional constraints on regional displacements, the current method could be adapted to have a spatially varying activation parameter. The method could be also be augmented by adding a time-varying activation model, such as an Eikonal model (Keener, 1991), to specify the spatially varying sequence of activation.

### 4.5. Regional Stress and Strain

From full-cycle simulations, active fibre stress and fibre stretch were plotted over 16 AHA regions in Figure 9 illustrating regional differences. In some DCM cases, fibre stretch in some regions was greater than one in early systole, signifying dilation. This may be due to regional systolic dysfunction in these cases. In general, DCM cases showed smaller magnitudes of fibre stretch than healthy and HCM groups, in line with typical systolic dysfunction marked in DCM (Hayashida et al., 1990). Fibre stretch, as opposed to circumferential and longitudinal stretch, could provide more direct measurements of how muscle fibres change with disease. Circumferential stretch measured from the models, plotted in Supplementary Figure 8, were comparable to circumferential strain measured from ultrasound in healthy individuals (Hurlburt et al., 2007; Leitman et al., 2010; Duan et al., 2012). However, model-derived longitudinal stretch was underestimated compared with longitudinal strain from ultrasound. Longitudinal strain is largely dependent on the defined fibre orientation and the model used to describe active contraction. In future, patient-specific fibres as well as constitutive models should be adapted to achieve physiological longitudinal strains. Stress and strain from personalised simulations such as these can provide valuable insights into cardiac function on an individual basis.

### 4.6. Limitations

In previous cardiac modelling studies, inverse methods have been used to estimate the unloaded geometry of the heart (Krishnamurthy et al., 2013; Wang Y. et al., 2020) which are dependent on the choice of material law, stiffness parameters and boundary conditions. In other studies, various points in the cardiac cycle have been used as the reference geometry including end-systole (Wang et al., 2009), early-diastole (Xi et al., 2013), and diastasis (Wang et al., 2018). However, physiologically, the heart is never in an entirely unloaded state. In early diastole, residual active stress may be present and in all phases of diastolic filling, the cavity pressure is never zero. Although passive parameter estimates have been shown to be minimally affected by changing the reference state from end-systolic to early-diastolic geometries (Hadjicharalambous et al., 2014b), the impact of the choice of reference state is examined further in Hadjicharalambous et al. (2021) and should be assessed in biventricular patient-specific modelling.

The personalised parameters in this study, e.g., *a*_0_, *a*_*f*_, α_*lv*_, and α_*rv*_, are all dependent on pressure estimates. If available, catheter measurements from within the LV cavity would enable accurate scaling of these parameters for each individual, and better certainty on model data. LV filling pressures can also be approximated with knowledge of the peak blood flow through the mitral valve as well as the mitral valve peak annular velocity (Nagueh et al., 1997). Given that the mitral valve annular velocity can be obtained in the current pipeline using the landmark predicted valve points, the additional acquisition of 4D flow MR imaging could provide measurements of peak blood flow, enabling appropriate personalisation of all parameters through non-invasive imaging. New methods, such as the use of microbubbles within the LV (Forsberg et al., 2005; Dave et al., 2012), may soon enable more accurate non-invasive cavity pressure measurements. Here, we demonstrate the feasibility of the personalised modelling method using standardised pressure data. If available, pressure data can easily be incorporated into the current pipeline.

## 5. Conclusions

This work presents a pipeline using neural networks for generating high quality biventricular models from standard MR cine data. A cohort of 12 individuals were used to demonstrate the pipeline in three different groups: healthy volunteers, DCM patients and HCM patients. Despite the varied morphology and motion of each case, the automated pipeline robustly allowed for determination of a unique passive material parameter describing the tissue anisotropy (γ) as well as two active scaling parameters controlling systolic contraction in the LV and RV (α_*lv*_ and α_*rv*_). The entire pipeline was run using only images from a typical clinical scan, demonstrating its potential to be applied to a large cohort of retrospective data. The use of neural networks along with the model fitting step significantly sped up the process for creating high-quality finite element models. cardiac cycle. This study demonstrates a pipeline that is suitable to model cardiac mechanics and estimate personalised parameters in a diverse cohort of individuals including healthy volunteers, DCM and HCM patients with varying morphologies.

## Data Availability Statement

The raw data supporting the conclusions of this article will be made available by the authors, without undue reservation.

## Ethics Statement

The studies involving human participants were reviewed and approved by London Bridge National Research Ethics Service. The patients/participants provided their written informed consent to participate in this study.

## Author Contributions

RM implemented the pipeline, created the tool for fibre field generation, and ran all simulations to estimate patient-specific material properties. EK developed both networks for MR labelling and landmark identification. CM developed the biventricular model fitting tool. TI collected the MR imaging data from HCM patients. AY gave input on the landmark network and revised the manuscript. DN was involved in supervision and collection of DCM and healthy volunteer imaging data. DN, RM, and EK were involved in writing the manuscript. All authors critically reviewed the manuscript.

## Funding

DN would like to acknowledge funding from Engineering and Physical Sciences Research Council (EP/N011554/1 and EP/R003866/1). This work was also supported by the Wellcome ESPRC Centre for Medical Engineering at King's College London (WT 203148/Z/16/Z) and the British Heart Foundation (TG/17/3/33406). AY would like to acknowledge funding from the National Heart, Lung and Blood Institute (NIH R01HL121754).

## Conflict of Interest

The authors declare that the research was conducted in the absence of any commercial or financial relationships that could be construed as a potential conflict of interest.

## Publisher's Note

All claims expressed in this article are solely those of the authors and do not necessarily represent those of their affiliated organizations, or those of the publisher, the editors and the reviewers. Any product that may be evaluated in this article, or claim that may be made by its manufacturer, is not guaranteed or endorsed by the publisher.

## Footnotes

^1^http://www.simmetrix.com/.
