# Supplementary material for: An Implementation of Patient-Specific Biventricular Mechanics Simulations With a Deep Learning and Computational Pipeline
Source: Front Physiol. 2021 Sep 16;12:716597. doi: 10.3389/fphys.2021.716597 (PMC8481785; doi:10.3389/fphys.2021.716597)
Supplement: Supplementary file 1 [file Data_Sheet_1.pdf]

Supplementary Material

1 FIBRE ORIENTATION

Fibre angles within the LV were set to vary from -60° to +60° from the epicardium to the endocardium. In the RV, fibres transitioned from -25° on the epicardium to 90°, or longitudinal, on the endocardium. Within the septum, fibres vary from 60° at the LV endocardium to 0° at the midwall to 90° in the RV (see Figure S1). The fibre angles prescribed at valve annuli are shown in Table S1. The angles prescribed were based on measurements from high-resolution ex-vivo diffusion images acquired in porcine hearts. Moving towards the valve annuli, angles transition rapidly to the prescribed valve angles.

Table S1. Fibre angles in degrees at valve annuli.

Valve	Endocardium	Epicardium
Mitral	60	0
Aortic	0	0
Tricuspid	0	-25
Pulmonary	90	0

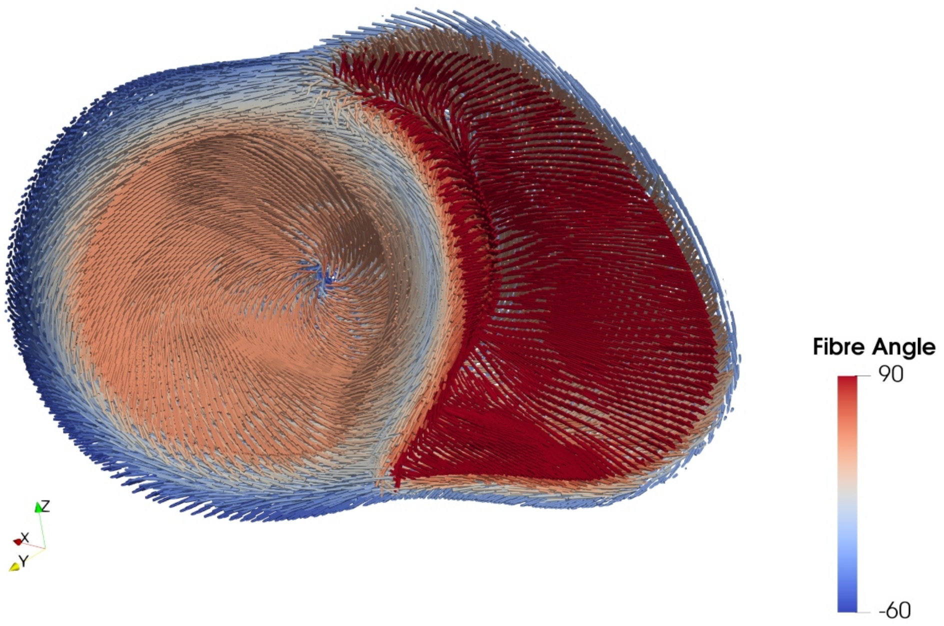

Figure S1. Short axis cut view to illustrate fibre vector variation in the septum going from 60° in the LV to 0° in the midwall to 90° in the RV.

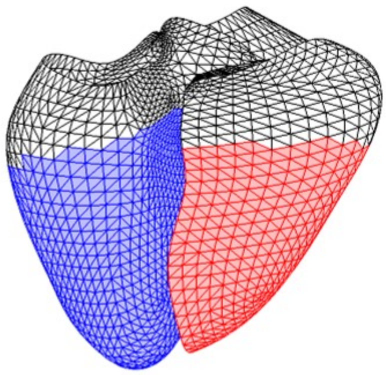

Figure S2. The endocardial surfaces meshes for case h2 are shown at the end-diastolic state illustrating regions used to calculate volume metrics for both the LV (blue) and RV (red).

2 VOLUME MEASUREMENTS

In addition to the values presented in Table 2 , clinical metrics, such as cavity volumes and ejection fraction, were also calculated from the short-axis segmentations from the neural network. Both values are shown here for comparison. However, the volumes are calculated differently and, therefore, do not represent precisely the same region of the heart. For example, the volumes (V_s) measured from the segmentations were calculated using a midpoint approximation:

$$V_s = \frac{h}{2} \sum_{i=1}^{N_s} (A_i + A_{i+1}) \tag{S1}$$

where h is the slice spacing, N_s is the number of slices and A is the cavity area. For the model geometry, the volumes within the mesh were calculated by cutting the cavities at a plane perpendicular to the long axis of each ventricle such that, at the end-systolic time point, all nodes in the valves were above that plane. The nodes/elements below that plane are then used to calculate the volume at all time points in the cardiac cycle. Therefore, the volumes listed in Table 2 represent the volume within a given region of tissue over time, tracking that tissue as it moves. This differs from the way that volumes are calculated from short-axis slices in that the short-axis planes are stationary over the cardiac cycle. The longitudinal motion of the heart means that, at one time point, such as the end-diastolic state, a single short-axis slice may be located within the basal region of the left ventricle whereas, in the end-systolic state, the same slice may be located within the atrium. Therefore, this may cause discrepancies in not only cavity volume estimates but also the calculation of ejection fraction. An example view of the cut regions is shown in Figure S2. The closed cut surface mesh was used to calculate the volume within the interior region.

In addition to using fundamentally different methods for the volume calculation, another primary difference between the two methods of volume calculation is the region included in the volume calculation. To calculate the volumes from the model geometry, a plane is automatically chosen which is perpendicular to the long-axis such that no valve nodes are included in the cut cavity region. This region is chosen entirely independently of the short-axis image locations. Therefore, the volumes measured from short-axis segmentations may be greater or less than the model calculated volumes, depending on the location of the cut plane. In general, LV volumes measured from the models were greater than those measured from short-axis segmentations whereas RV volumes were less than those measured from segmentations, indicating that the RV cut plane lay below the basal-most short-axis slice.

Due to these differences, it is difficult to draw significant conclusions about the accuracy of either method. However, one previous study has compared similar short-axis volume calculation methods with a model surface fitting method. EF measured from short-axis slices has an uncertainty as high at 49% whereas EF derived from fitted surfaces was shown to have error less than 3% (O'Dell, 2019). Additionally, volumes measured from short-axis segmentations are sensitive to, not only the number, but also location of slices.

Table S2. Functional indices: end-diastolic volume (EDV), end-systolic volume (ESV), ejection fraction (EF). Values are shown which were derived from (a) the modelling pipeline (gray rows) and (b) the short-axis neural network segmentations (white rows).

	Left Ventricle			Right Ventricle		
Case	EDV (mL)	ESV (mL)	EF (%)	EDV (mL)	ESV (mL)	EF (%)
Healthy Volunteers						
**v1**	172.7	80.8	53.2	104.2	64.2	38.4
	162.6	88.8	45.4	183.5	132.2	45.4
**v2**	169.9	79.4	53.3	114.4	48.9	57.3
	149.7	71.4	52.3	162.3	102.1	52.3
**v3**	170.1	76.7	54.9	104.9	47.6	54.6
	140.6	81.4	42.1	175.9	125.4	42.1
**v4**	123.7	48.6	60.7	81.1	42.0	48.2
	92.4	43.1	53.4	138.9	85.1	53.4
DCM Patients						
**d1**	124.4	73.7	40.8	57.9	36.4	37.1
	106.4	65.0	38.9	70.2	43.5	38.9
**d2**	231.8	130.0	44.0	106.8	57.8	45.9
	204.6	133.4	34.8	167.6	114.6	34.8
**d3**	172.7	90.1	47.8	126.5	58.4	53.8
	182.3	101.9	44.1	161.4	95.0	44.1
**d4**	171.0	99.9	41.5	87.8	46.4	47.2
	175.4	111.2	36.6	142.9	106.0	36.6
HCM Patients						
**h1**	125.8	59.2	52.9	69.9	34.4	50.8
	85.51[a]	35.43	58.56	60.0	41.0	68.5
**h2**	105.8	35.1	66.8	65.2	31.7	51.4
	106.6	27.4	74.3	55.9	45.9	74.3
**h3**	134.9	55.3	59.1	87.0	44.1	49.3
	126.0	56.5	55.2	57.9	50.3	55.2
**h4**	113.7	41.0	63.9	98.4	44.8	54.5
	104.5	26.6	74.5	68.4	43.0	74.5

[a] The large discrepancy in cavity volumes for this case is due to the fact that the short-axis slices were only collected between the base and mid-ventricle, omitting the apical region in the short-axis volume calculation.

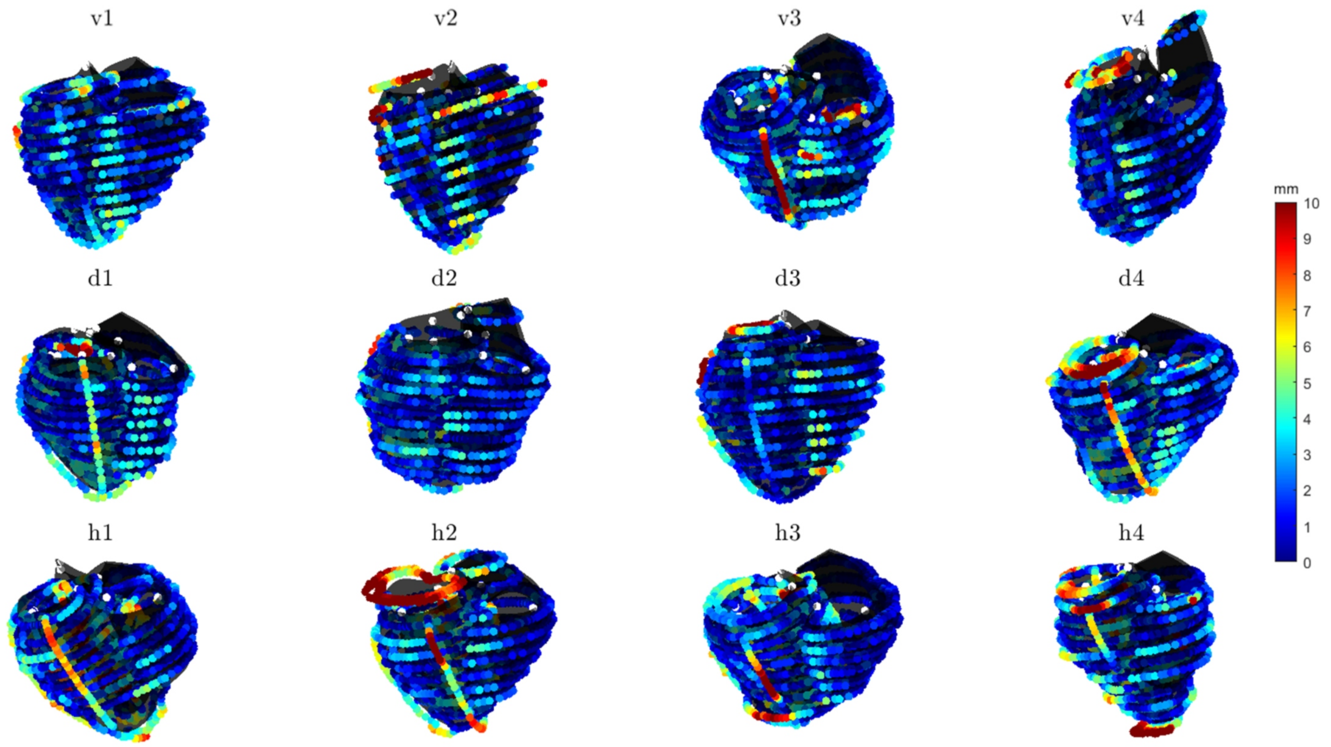

Figure S3. Each surface mesh is shown (black) with contour points derived from neural network segmentations. Errors represent the distance (mm) from each contour point to its corresponding model surface.

3 MODEL FITTING ERRORS

A template mesh was fit to contour points generated from neural network segmentations. Figure S3 illustrates the models fit to the end-systolic time point for each case. Contour points are coloured by the distance from the point to its corresponding surface (e.g. the distance from epicardial contours to the epicardial surface).

4 WALL THICKNESS

Bullseye plots of wall thickness (mm) in the left ventricle illustrate regional differences, averaged over each patient group. It can be seen that, particularly during diastole, the HCM group had higher wall thickness in the anterior septal region. The DCM group demonstrated minimal changes in wall thickness betweed ED and ES, marking reduced systolic function.

5 MESH METRICS

The number of nodes, number of elements, and mean element quality ($\pm$ one standard deviation) are shown in Table 2 . Due to the standardized process for generating meshes, the element quality shows little variation between cases. However, where there were more rapid shape changes, more elements were needed to capture the shape accurately.

6 INFLATED MODELS

Figure S5 illustrates the relative capability of the pipeline to predict the end-diastolic state through biomechanical simulations. The outline represents the final inflated state of the model. Although some

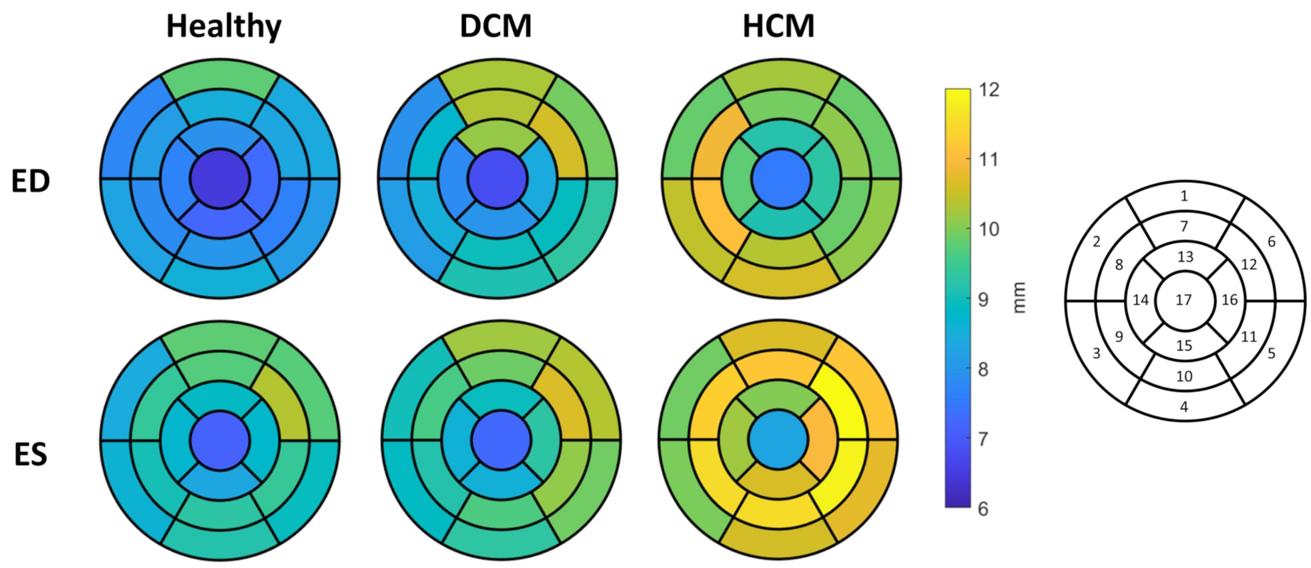

Figure S4. Bullseye plots illustrating mean wall thickness for healthy volunteers, DCM patients and HCM patients.

Table S3. Mesh metrics: number of nodes, number of elements and element quality (q). Element quality q was computed as $q = 3r$, r being the ratio of inradius to circumradius.

Case	Nodes	Elems	q ± std
v1	71,254	335,066	0.818 ± 0.092
v2	57,113	266,799	0.819 ± 0.093
v3	74,663	354,513	0.822 ± 0.087
v4	55,062	254,645	0.823 ± 0.089
d1	61,966	294,717	0.825 ± 0.082
d2	88,179	422,512	0.819 ± 0.084
d3	69,565	332,944	0.825 ± 0.080
d4	77,878	375,714	0.823 ± 0.080
h1	88.376	440,004	0.808 ± 0.078
h2	49,883	229,118	0.829 ± 0.085
h3	70,470	333,821	0.825 ± 0.080
h4	66,520	328,136	0.817 ± 0.076

features of the shapes do not resemble those in the images, such as near the RV insertion points, the LV shapes are generally well approximated using the biomechanical simulation.

7 IMPACT OF RIGHT VENTRICLE ON OBJECTIVE FUNCTION

When no epicardial constraints are used on the RV boundary, volumetric inflation results in a consistent change in diameter throughout the RV. However, it can be seen in Figure S6 that, under physiological conditions, the right heart deformation is constrained and the final state is less round. Therefore, appropriate epicardial constraints are needed in future studies to accurately model RV behaviour.

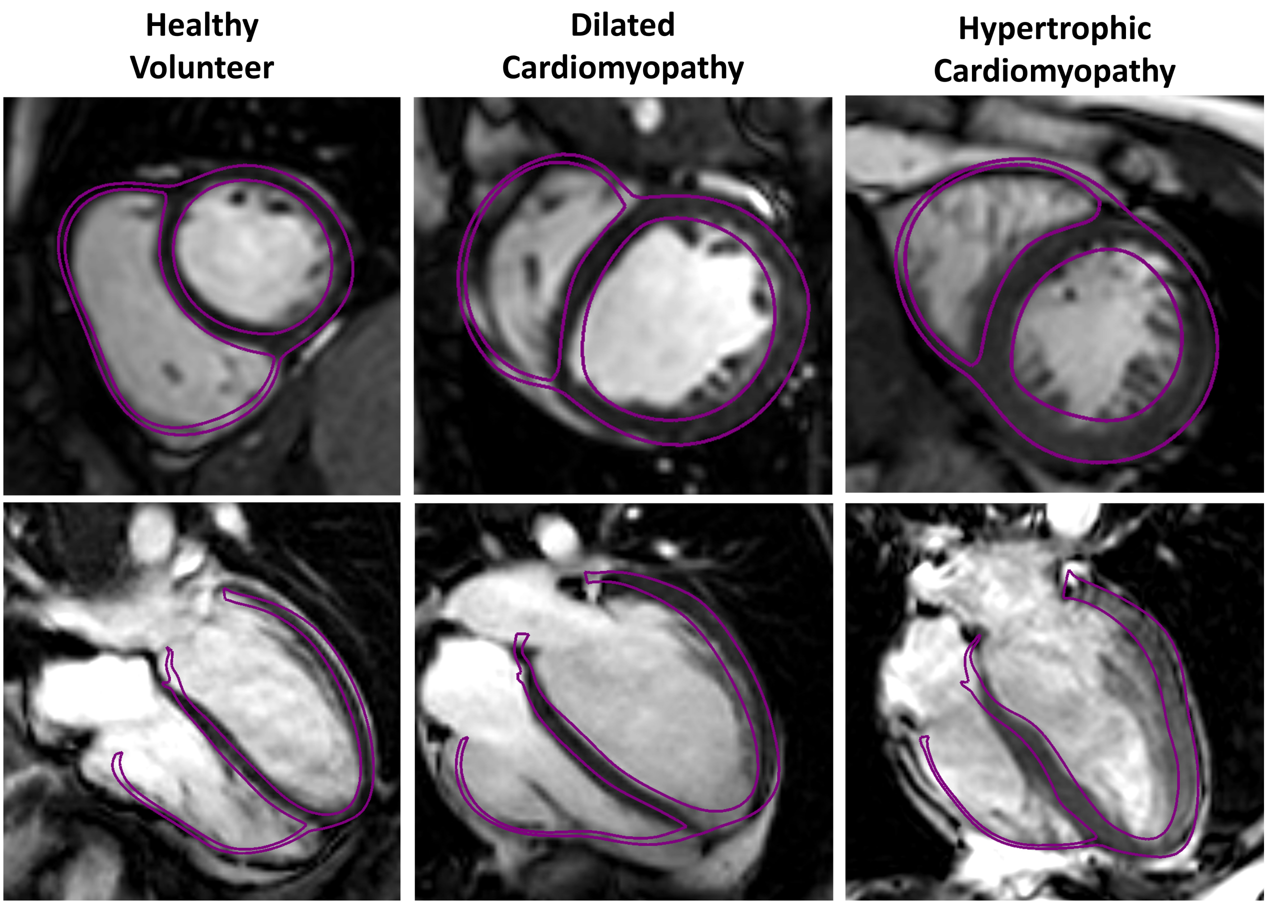

Figure S5. Three representative cases (including one healthy volunteer (v4), one DCM patient (d3) and one HCM patient (h4)) are shown below with final inflated models at the end-diastolic state overlain on the short-axis and 4CH long-axis images at end-diastole.

Figure S7 illustrates objective functions when using contour points from the LV only (including the endocardial, epicardial and RV septum) or the LV and RV free wall contours. Including the RV free wall contours resulted in higher objective function errors and, in some cases, no local minimum. Therefore, although the inclusion of RV mechanics and valve boundary conditions is important for constraining physiological motion of the LV, the deformation of the RV is largely impacted by epicardial boundary conditions due to its thin walled structure. Constraining RV volumes alone did not result in accurate deformation on the right side of the heart. Therefore, these contour points were excluded from the final objective function.

8 CIRCUMFERENTIAL AND LONGITUDINAL STRETCH

Circumferential and longitudinal stretch in the 16 AHA regions were calculated for each case. In general, the DCM cases demonstrated smaller circumferential stretch than the healthy volunteers. There were no significant differences between longitudinal stretch in the three groups.

9 REQUIRED TIME AND RESOURCES

The approximate time required for each step of the model preparation pipeline is shown in Table S5 along with an approximate time for completing the same steps manually. The biomechanical simulations, including the passive parameter estimation and the full-cycle simulations, were run on a supercomputer

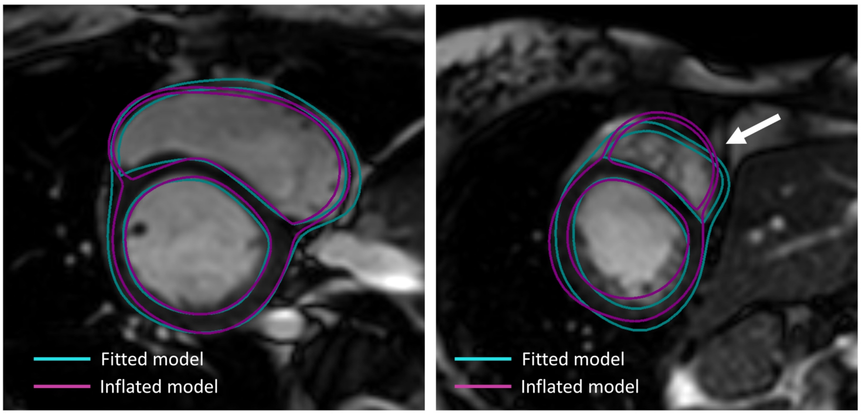

Figure S6. Figure showing two short-axis slices from a single case (d3) with outlines of the model fit to the neural network segmentations at the end-diastolic state (cyan) as well as the model inflated to the end-diastolic state (magenta).

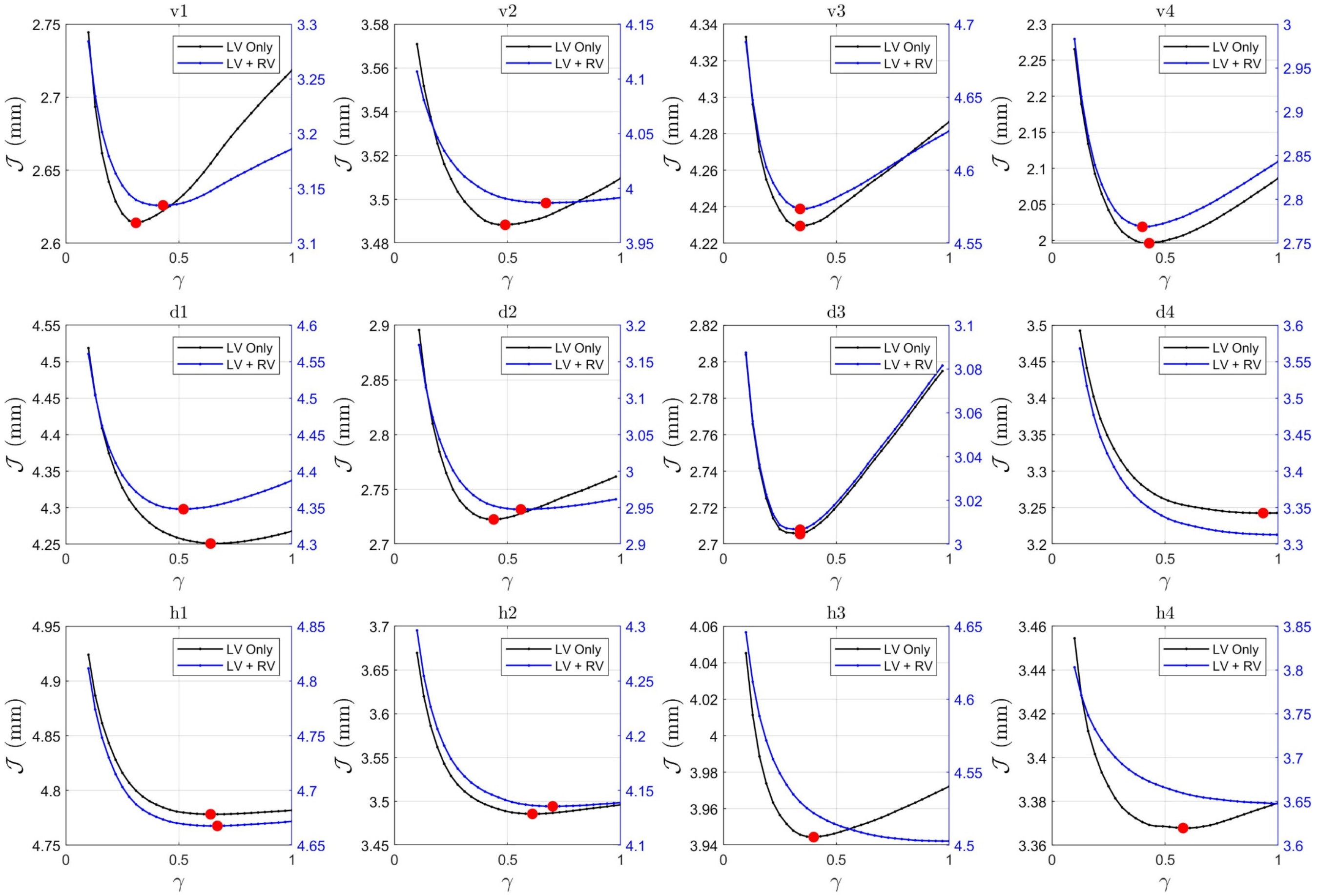

Figure S7. Objective functions ($\mathcal{J}$) illustrating optimal values of γ for all cases when utilising contour points from either the LV only (black) or LV and RV (blue).

with 32 cores (Intel(R) Xeon(TM) E7-8837 2.6 GHz). The passive parameter estimation and full-cycle simulations took approximately 135 minutes and 310 minutes, respectively. The primary advantage of this pipeline lies in the steps to set up the model for the simulations, which are rapid and require no manual intervention.

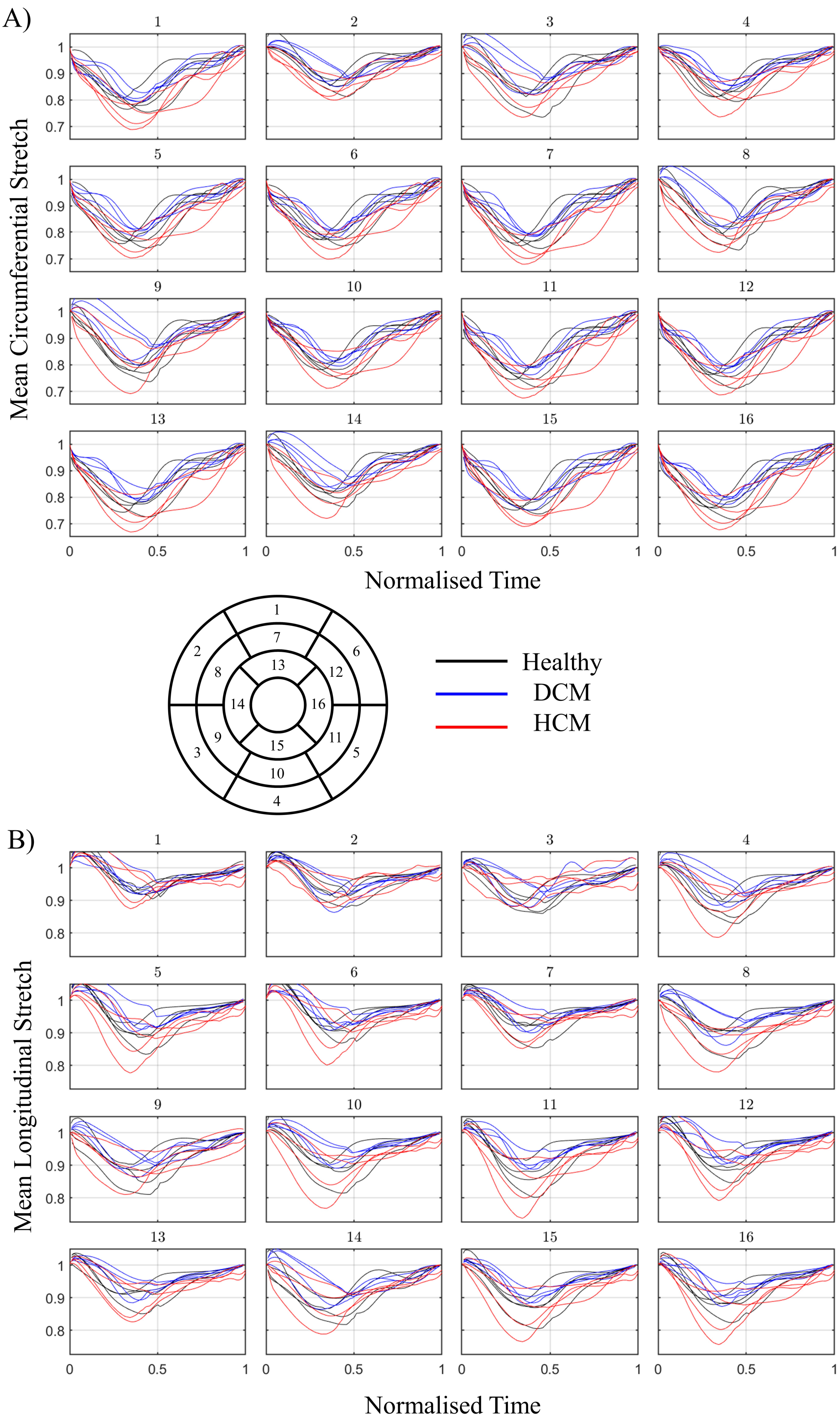

Figure S8. (A) Mean circumferential stretch and (B) mean longitudinal stretch over the cardiac cycle in 16 AHA regions of the LV for healthy (black), DCM (blue) and HCM (red) groups.

Table S4. Peak circumferential and longitudinal strain listed for each AHA segment. Mean ± one standard deviation are shown for each group. * indicates statistically significant differences when compared to the healthy volunteers ($p < 0.05$).

	Circumferential			Longitudinal		
Segment	Vol	DCM	HCM	Vol	DCM	HCM
1	22.4 ± 2.4	19.6 ± 1.6	26.8 ± 4.2 *	-8.1 ± 1.3	-6.9 ± 1.8	-9.1 ± 3.0
2	15.3 ± 2.6	13.0 ± 1.5	16.9 ± 3.5	-9.6 ± 1.7	-8.7 ± 3.5	-9.2 ± 3.6
3	19.9 ± 4.6	14.9 ± 3.3	20.3 ± 5.7	-12.06 ± 2.0	-10.1 ± 2.2	-8.7 ± 4.1
4	18.8 ± 1.5	14.6 ± 1.5 *	19.9 ± 5.4	-13.2 ± 3.5	-10.1 ± 2.1	-13.0 ± 6.0
5	24.3 ± 1.2	19.7 ± 1.3 *	25.0 ± 4.5	-11.7 ± 3.6	-8.0 ± 2.0	-14.6 ± 5.9
6	23.4 ± 1.2	20.0 ± 1.4 *	25.0 ± 4.2	-9.8 ± 2.5	-7.3 ± 2.1	-12.8 ± 5.8
7	24.7 ± 1.2	21.1 ± 0.5 *	27.8 ± 4.8 *	-9.8 ± 3.3	-7.5 ± 2.8	-10.1 ± 4.5
8	22.0 ± 3.7	16.7 ± 2.0 *	22.7 ± 4.7	-11.6 ± 4.2	-9.5 ± 3.6	-14.5 ± 6.0
9	23.1 ± 2.7	16.1 ± 3.8 *	21.4 ± 7.1	-13.9 ± 4.1	-11.1 ± 1.9	-12.8 ± 5.4
10	22.2 ± 2.4	19.0 ± 1.2	23.0 ± 5.84	-12.7 ± 4.0	-8.3 ± 2.5	-14.1 ± 7.1
11	25.8 ± 2.1	21.8 ± 1.4 *	27.0 ± 5.2	-13.7 ± 4.8	-9.6 ± 2.6	-17.1 ± 7.9
12	25.2 ± 2.6	21.5 ± 1.2 *	26.3 ± 4.6	-11.3 ± 3.1	-8.0 ± 2.3	-13.9 ± 6.5
13	24.8 ± 2.3	20.0 ± 2.1 *	27.3 ± 6.0	-10.4 ± 3.0	-8.5 ± 3.2	-12.1 ± 5.5
14	20.2 ± 2.6	15.3 ± 2.0 *	20.9 ± 5.7	-14.4 ± 3.4	-11.1 ± 2.2	-14.0 ± 5.6
15	25.0 ± 1.2	22.0 ± 2.0 *	27.4 ± 4.4	-14.0 ± 3.8	-10.1 ± 2.1	-15.5 ± 7.1
16	25.1 ± 2.3	21.1 ± 1.2 *	26.9 ± 5.6	-13.0 ± 3.2	-9.5 ± 2.8	-16.1 ± 7.4

Table S5. Approximate time required for both the manual and automated pipeline.

Step	Manual	Automated Pipeline	Automated Pipeline Resources
Segmentation (30 frames x 10 SA slices, 30 frames x 3 LA views)	780 mins	0.26 mins	Intel(R) Core(TM) i7-5930K, CPU @ 3.50GHz (Python)
Landmark identification (30 frames x 3 LA views)	20 mins	0.06 mins	Intel(R) Core(TM) i7-5930K, CPU @ 3.50GHz (Python)
BV model fitting (30 frames), Volumetric meshing (ES only), Surface labelling	240 mins	25 mins	Intel(R) Core(TM) i7-8750H CPU @ 2.20GHz (Python, SimModeler)

REFERENCES

O'Dell WG. Accuracy of Left Ventricular Cavity Volume and Ejection Fraction for Conventional Estimation Methods and 3D Surface Fitting. *Journal of the American Heart Association* **8** (2019).
